# Effect of a Multisectoral Agricultural Intervention on HIV Health Outcomes Among Adults in Kenya: A Cluster Randomized Clinical Trial

**DOI:** 10.1001/jamanetworkopen.2022.46158

**Published:** 2022-12-12

**Authors:** Craig R. Cohen, Elly Weke, Edward A. Frongillo, Lila A. Sheira, Rachel Burger, Adrienne Rain Mocello, Pauline Wekesa, Martin Fisher, Kate Scow, Harsha Thirumurthy, Shari L. Dworkin, Starley B. Shade, Lisa M. Butler, Elizabeth A. Bukusi, Sheri D. Weiser

**Affiliations:** 1 Department of Obstetrics, Gynecology & Reproductive Sciences, University of California, San Francisco; 2 Centre for Microbiology Research, Kenya Medical Research Institute, Nairobi; 3 Arnold School of Public Health, University of South Carolina, Columbia; 4 Department of Medicine, University of California, San Francisco; 5 KickStart International, San Francisco, California; 6 Department of Land, Air and Water Resources, University of California, Davis; 7 Department of Medical Ethics and Health Policy, Perelman School of Medicine, University of Pennsylvania, Philadelphia; 8 School of Nursing and Health Studies, University of Washington-Bothell, Bothell; 9 Department of Epidemiology and Biostatistics, University of California, San Francisco; 10 Institute for Collaboration on Health, Intervention, and Policy, University of Connecticut, Storrs

## Abstract

**Importance:**

Food insecurity and HIV health outcomes are linked through nutritional, mental health, and health behavior pathways.

**Objective:**

To examine the effects of a multisectoral agriculture and livelihood intervention on HIV viral suppression and nutritional, mental health, and behavioral outcomes among HIV-positive adults prescribed antiretroviral therapy (ART).

**Design, Setting, and Participants:**

This cluster randomized clinical trial was performed in 8 pairs of health facilities in Kenya. Participants were 18 years or older, living with HIV, and receiving ART for longer than 6 months; had moderate to severe food insecurity; and had access to arable land and surface water and/or shallow aquifers. Participants were followed up every 6 months for 24 months. Data were collected from June 23, 2016, to June 13, 2017, with follow-up completed by December 16, 2019. Data were analyzed from June 25 to August 31, 2020, using intention-to-treat and per-protocol methods.

**Interventions:**

A loan to purchase a human-powered irrigation pump, fertilizer, seeds, and pesticides combined with the provision of training in sustainable agriculture and financial literacy.

**Main Outcomes and Measures:**

The primary outcome was the relative change from baseline to the end of follow-up in viral load suppression (≤200 copies/mL) compared between study groups using difference-in-differences analyses. Secondary outcomes included clinic attendance, ART adherence, food insecurity, depression, self-confidence, and social support.

**Results:**

A total of 720 participants were enrolled (396 women [55.0%]; mean [SD] age, 40.38 [9.12] years), including 366 in the intervention group and 354 in the control group. Retention included 677 (94.0%) at the 24-month visit. HIV viral suppression improved in both groups from baseline to end of follow-up from 314 of 366 (85.8%) to 327 of 344 (95.1%) in the intervention group and from 291 of 353 (82.4%) to 314 of 333 (94.3%) in the control group (*P* = .86). Food insecurity decreased more in the intervention than the control group (difference in linear trend, −3.54 [95% CI, −4.16 to −2.92]). Proportions of those with depression during the 24-month follow-up period declined more in the intervention group (from 169 of 365 [46.3%] to 36 of 344 [10.5%]) than the control group (106 of 354 [29.9%] to 41 of 333 [12.3%]; difference in trend, −0.83 [95% CI, −1.45 to −0.20]). Self-confidence improved more in the intervention than control group (difference in trend, −0.37 [95% CI, −0.59 to −0.15]; *P* = .001), as did social support (difference in trend, −3.63 [95% CI, −4.30 to −2.95]; *P* < .001).

**Conclusions and Relevance:**

In this cluster randomized trial, the multisectoral agricultural intervention led to demonstrable health and other benefits; however, it was not possible to detect additional effects of the intervention on HIV clinical indicators. Agricultural interventions that improve productivity and livelihoods hold promise as a way of addressing food insecurity and the underpinnings of poor health among people living with HIV in resource-limited settings.

**Trial Registration:**

ClinicalTrials.gov Identifier: NCT02815579

## Key Points

**Question:**

Does a multisectoral, climate-adaptive, agricultural livelihood intervention improve health outcomes for individuals living with HIV and food insecurity?

**Findings:**

In this cluster randomized clinical trial of 720 participants, the intervention did not lead to improvements in HIV viral suppression for adults living with HIV and receiving antiretroviral therapy. Viral suppression approached the UNAIDS goal of at least 95% in both study groups in the setting of widespread test and treatment policies launched during the study, and the intervention led to improvements of predefined secondary outcomes, including food security, mental health, self-confidence, and social support.

**Meaning:**

In the setting of high-quality HIV service delivery, a multisectoral agricultural and livelihood intervention did not affect viral suppression.

## Teaser

This cluster randomized clinical trial assesses whether a multisectoral agricultural livelihood intervention improves viral load suppression, reduces food insecurity, and improves nutrition, mental health, and empowerment indicators among people living with HIV in sub-Saharan Africa.

## Introduction

Food insecurity, defined as “the limited or uncertain availability of nutritionally adequate, safe foods or the inability to acquire personally acceptable foods in socially acceptable ways,”^1^ is highly prevalent in sub-Saharan Africa. In 2020, approximately 752 million people in the region—66.2% of the total population—experienced moderate to severe food insecurity, with a higher prevalence among persons living with HIV (PLHIV).^2,3,4^ Despite progress in reducing world hunger and malnutrition, food insecurity has been exacerbated by the COVID-19 pandemic^5,6^ and is expected to rise further in coming decades owing to global geopolitical supply disruptions, climate change, and environmental degradation.^7^ HIV/AIDS and food insecurity are intertwined through biological, behavioral, and socioeconomic pathways that can worsen immunologic and virologic responses and cause treatment interruptions and nonadherence.^8^ Among PLHIV, food insecurity is associated with declines in physical health status,^9^ decreased viral suppression,^9,10,11^ worse immunologic status,^9,10,12^ increased incidence of serious illness,^12,13^ and increased mortality.^11^ The bidirectional linkages between HIV/AIDS and food insecurity, in which each heightens vulnerability to and increases the severity of the other, are often amplified by weak health care systems, unsustainable agricultural practices, and entrenched poverty.

To ensure that food insecurity does not compromise the scale-up toward universal access to effective HIV care, global health agencies have recommended integrating sustainable food production strategies into HIV/AIDS programming.^14^ Interventions among PLHIV that aim to improve nutritional status through food assistance and macronutrient supplementation have yielded improved HIV-related outcomes, including better treatment adherence.^15,16^ Although these strategies address the malnutrition risks associated with food insecurity, they have limited scalability and fail to address root causes such as poverty, disrupted livelihoods, and reduced agricultural yields driven by climate change. Structural interventions that target these underlying drivers may result in sustained improvements in both food security and health outcomes. In sub-Saharan Africa, where agriculture accounts for more than half of the total workforce and 15% of the gross domestic product, reaching as high as 50% in some countries,^17^ combined agricultural and livelihood interventions may be especially well suited to sustainably improving food security and HIV-related health outcomes for PLHIV. The previously published *Shamba Maisha* (“Farming for Life” in Kiswahili) pilot cluster randomized clinical trial (RCT) of a multisectoral agricultural livelihood intervention (140 PLHIV randomized to intervention or control) in western Kenya^18^ found that participants in the intervention group had improvements in food security (3.6 scale points higher; *P* < .001), CD4 cell counts (165 cells/mm^3^ greater; *P* < .001), and viral suppression (odds ratio, 7.6 [95% CI, 2.2-26.8]; *P* = .002) compared with controls.

In the present study, we performed a cluster RCT to test the hypothesis that the *Shamba Maisha* intervention would improve viral load suppression, reduce food insecurity, and improve nutrition, mental health, and empowerment indicators among PLHIV. We aimed to determine cluster-level effects of the intervention on HIV clinical outcomes in Kenya as well as effects on intermediate outcomes in our evidence-based causal framework.

## Methods

### Trial Design

We performed a pair-matched cluster RCT in which the clusters were health facilities in Kisumu, Homa Bay, and Migori counties in Kenya. Pairs were matched based on size of the facility, geography defined by subcounty, primary sources of water for irrigation, and access to markets. Adult patients (18 years and older) with moderate to severe food insecurity who received HIV care and treatment at an enrolled facility (8 facilities in the intervention group and 8 in the control group) were eligible for screening and enrollment into the trial. A copy of the trial protocol is found in Supplement 1. We received ethics approval from the Kenya Medical Research Institute and the Institutional Review Board of the University of California, San Francisco. Participants provided written informed consent. This report follows the Consolidated Standards of Reporting Trials (CONSORT) reporting guideline for cluster RCTs (additional details can be found in the eMethods in Supplement 2).

### Intervention

Informed by the results of the pilot study,^18^ we developed and tested a multisectoral cluster-level intervention (*Shamba Maisha*) that aimed to address root causes of food insecurity and poor health in the region, namely a lack of irrigation compounded by unpredictable rainfall patterns and increasing frequency of drought and flooding owing to worsening climate change. The intervention consisted of (1) a market interest loan (approximately $175 US) from Equity Bank Kenya, Ltd, in Nairobi required to purchase agricultural implements; (2) the agricultural implements purchased with the loan, including a human-powered water pump (Super MoneyMaker; KickStart International), seeds, fertilizers, and pesticides; and (3) education in financial management and sustainable and regenerative farming practices (using 8 small-group didactic and discussion sessions and hands-on skills learning during the first 3 months of enrollment). Individuals in the control group were offered a similar intervention at the conclusion of their participation in the trial.

### Outcomes

The primary outcome was HIV RNA viral load suppression (measured as copies per milliliter in blood samples), which was assessed every 6 months among all study participants. For analysis of the primary outcome, we compared the proportion of participants with viral suppression, defined as less than or equal to 200 copies/mL at baseline, with the end of follow-up by study group.

The secondary outcomes were (1) food security measured via the Household Food Insecurity Access Scale^19^; (2) CD4 cell count; (3) hospitalizations during the past 6 months; (4) proportion with an AIDS-defining condition; (5) nutritional status, represented by body mass index (BMI; calculated as weight in kilograms divided by height in meters squared); (6) self-reported antiretroviral therapy (ART) adherence assessed by questionnaire and visual analog scale; (7) missed clinic visits; (8) depressive symptoms, measured via the Hopkins Symptom Checklist for Depression (with a value of ≥1.75 consistent with positive screening findings for symptoms of depression)^20^; (9) social support, measured using a modified version of the Duke University–University of North Carolina Functional Support Questionnaire consisting of questions related to perceived emotional and instrumental support^21^; and (10) self-confidence, measured via a 3-item scale.^22^ These scales have all been adapted in sub-Saharan Africa and/or were used in the pilot study.^18^

### Sample Size

We used data from our pilot study to estimate the sample size for the key outcomes: changes from months 0 to 24 in viral load suppression (primary outcome variable) and in food insecurity score (key mediating variable). We assumed that the SD values in this study would be similar to those in the pilot study^18^ because the populations had similar geographic and demographic characteristics. To be conservative, we assumed a coefficient of variation due to clustering of 0.150, ignoring the matched pairs.^23^ For 2-sided testing at α = .05 in a longitudinal analysis, a sample of 8 health facilities per group with 44 enrolled participants per health facility (total enrollment of 352 per group) would provide 80% power to detect an important clinical difference of 0.138 between intervention and control groups in the proportion with viral suppression from months 0 to 24 (primary outcome).^23^

### Randomization

Health facilities in Kisumu, Homa Bay, and Migori counties that provided HIV care and treatment were evaluated for inclusion in the trial and were considered if they had at least 350 active patients with HIV receiving ART and without a high probability of contamination via participant movement between intervention and control study populations. We selected 16 health facilities consisting of 8 well-matched pairs based on facility type (ie, subcounty hospital, health center, or dispensary); geography, defined by subcounty, soil type, primary source of water for irrigation (ie, lake, river, or stream; shallow wells); and access to markets. Facilities were randomized within pairs to intervention or control groups using random numbers computer generated by the study statistician (E.A.F.).

### Enrollment of Participants

**Figure 1.**
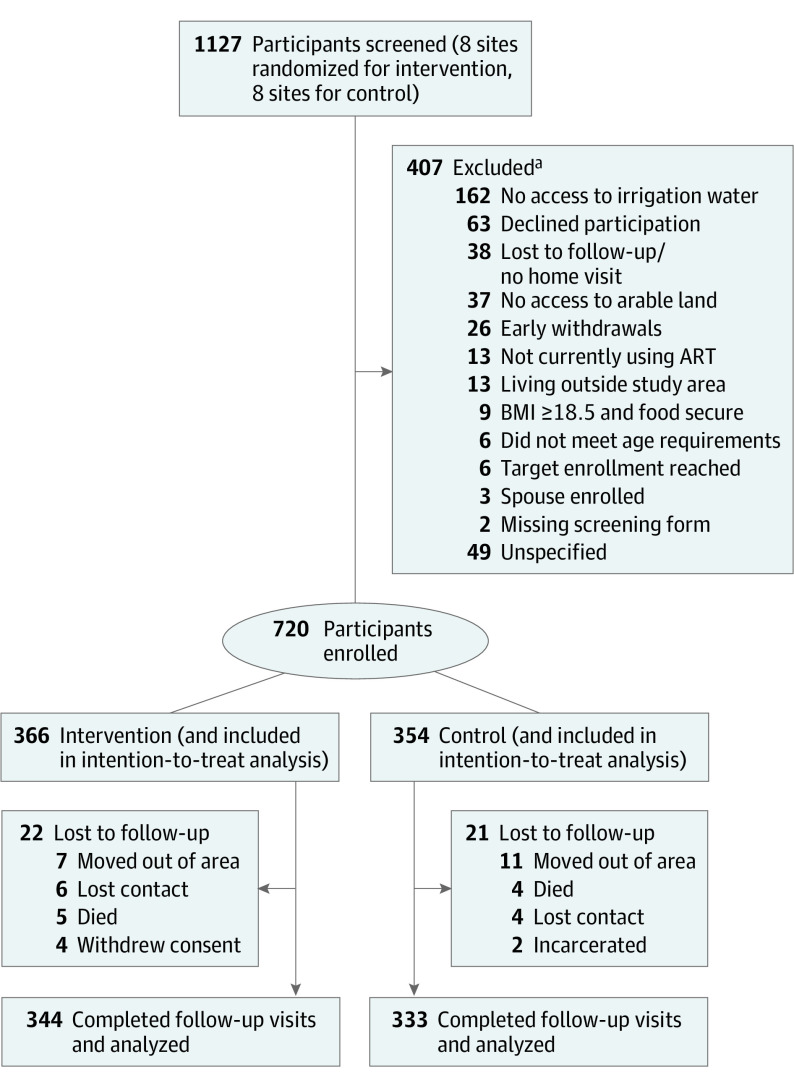
Study Flow Diagram ART indicates antiretroviral therapy; BMI, body mass index (calculated as weight in kilograms divided by height in meters squared). ^a^Some participants may have been excluded for more than 1 reason.

Screening and enrollment were performed from June 23, 2016, through June 13, 2017 (Figure 1), with the final follow-up visit 2 years later completed by December 16, 2019. Data on sex were self-reported. Ethnicity data were captured during research, and all participants were Kenyan in this study. We recruited through organized meetings held at each health facility. We conducted home visits among interested and potentially eligible individuals to verify that each participant had access to agricultural land and to surface water or a shallow, hand-dug well.

Across all intervention and control health facilities, we enrolled 44 to 55 persons per health facility who were currently receiving HIV care. Eligibility criteria consisted of (1) adults living with HIV and currently receiving ART for longer than 6 months, (2) 18 to 60 years of age, (3) belonging to a patient support group or indicating willingness to join one, (4) having access to arable land and water for irrigation (eg, lakes, rivers, ponds, or shallow wells), (5) evidence of moderate to severe food insecurity based on the Household Food Insecurity Access Scale and/or malnutrition (BMI <18.5) based on medical records during the year preceding recruitment, (6) agreement to save the required loan down payment (≤2000 Kenyan schillings [approximately $20 US]), and (7) ability to speak Dholuo, Kiswahili, or English. Persons with inadequate cognitive and/or hearing capacity to complete planned study procedures were excluded from enrollment. We implemented a viral load scale-up clinical facility readiness assessment^24^ between February 1, 2019, and April 30, 2020, at each health facility to help control for any potential cluster-level differences in ART prescribing practices that could affect viral suppression (eMethods in Supplement 2).

In anticipation of the approval and receipt of the asset loan, each intervention participant was immediately enrolled in a savings program and joined 1 of 3 patient support groups at each facility. These groups were used for provision and administration of individual loans as well as for training. At control facilities, participants were enrolled and offered the intervention at the end of 2 years of follow-up.

Each participant was asked to provide written informed consent for study procedures that occurred at baseline and at 6-month intervals for (1) collection of data via household and clinic-based surveys, (2) measurements of BMI and middle upper arm circumference to assess nutritional status, (3) collection of blood samples to measure HIV viral loads and CD4 counts twice per year, and (4) data abstraction from medical records. All intervention and control participants were reimbursed for their transportation and time according to local standards.

### Blinding

Owing to the nature of the intervention, participants and the research assistants collecting data could not be blinded to study group, but the laboratory staff performing viral load and CD4 testing were blinded to study group. The study team trained staff to implement data ascertainment consistently across both study groups to reduce bias, including the Hawthorne effect within the control group. Data management staff, all investigators (C.R.C., L.A.S., R.B., A.R.M., E.A.B., and S.D.W.), and the study biostatistician (E.A.F.) remained blinded to study group until after the database was frozen for analysis after data cleaning procedures and finalization of the statistical analysis plan (Supplement 1).

### Statistical Analysis

Data were analyzed from June 25 to August 31, 2022, using intention-to-treat and per-protocol methods. Distributions of all continuous outcome variables were examined. Intention-to-treat analyses were performed with all 720 enrolled participants using 3-level, mixed-effect, linear or logistic regression models estimated with maximum likelihood for all longitudinal variables. These mixed models were equivalent to repeated-measures models. Pairs, groups, visits, and the interactions between groups and visits were fixed effects. Clusters, individuals, and visits within individuals were modeled as random effects. Including or not including the pairs in analyses did not affect any results, so results were reported from models without the pair matching. Each of the 4 follow-up visits were compared with the first visit. Correspondingly, 4 interaction terms estimated the difference between study groups in changes from visit 1 to each follow-up visit. To determine the intervention effect, we compared trends in outcomes between the intervention and control groups using difference-in-differences regression. Linear contrasts among the group-by-visit interaction terms were used to estimate intervention effects for outcomes. For viral load suppression, the linear contrast was the interaction of group with visits 1 and 5 only, thus estimating the difference between groups in the change from visit 1 to visit 5. For all other outcomes, the linear contrast was the interaction of group and the linear trend over all visits, thus estimating the difference between groups in trend (ie, change) over visits and expressed as the trend for 24 months. We performed subgroup analyses to evaluate whether intervention effects differed by sex. Tests were 2-sided, and *P* < .05 indicated statistical significance. No adjusted analysis or adjustments for multiplicity were performed. For a per-protocol analysis of viral suppression, we included all 354 control participants and the 216 intervention participants (59.0%) who obtained the agricultural inputs after saving the down payment and attended at least 6 of 8 agriculture trainings sessions and 1 of 2 financial literacy trainings. We used Stata, version 16 (StataCorp LLC) for analyses and R Studio, version 4.0.5 (R Foundation for Statistical Computing) to create figures.

## Results

### Participant Screening, Enrollment, and Retention

**Figure 2.**
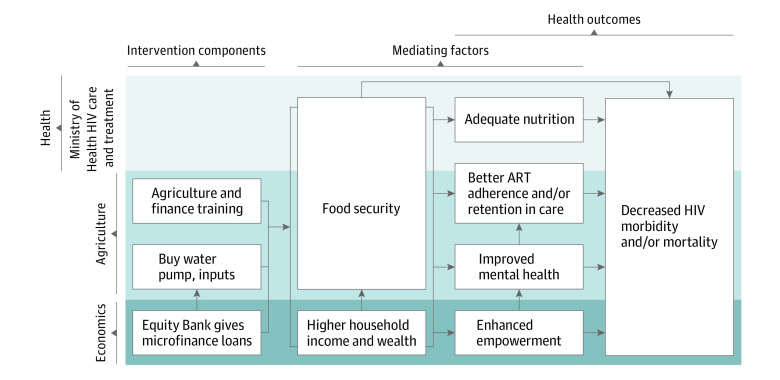
Intervention Theory of Change ART indicates antiretroviral therapy.

A total of 720 participants (396 women [55.0%] and 324 men [45.0%]; mean [SD] age, 40.38 [9.12] years) were included in the analysis. In the intervention group, we screened 606 PLHIV and enrolled 366 (60.4%); 216 (35.6%) were found ineligible before enrollment, and 24 (4.0%) were withdrawn before receipt of any study activities. Among 521 individuals screened at control facilities, 354 (67.9%) were enrolled; 165 (31.7%) were ineligible and 2 (0.4%) were early withdrawals. Sociodemographic factors were generally similar by study group; in the control group, the mean (SD) age was 40.4 (9.3) years with 194 women (54.8%) and 160 men (45.2%); in the intervention group, the mean (SD) age was 40.3 (8.9) years with 202 women (55.2%) and 164 men (44.8%) (Table 1). The 24-month study visits were completed by 344 participants (94.0%) in the intervention group and 333 (94.1%) in the control group. The study flow diagram is shown in Figure 1 and the causal framework is shown in Figure 2.

**Table 1.** Baseline Characteristics of the *Shamba Maisha* Study Population by Study Group

Characteristic	Study group^a^	
	Control (n = 354)	Intervention (n = 366)
Sex		
Women	194 (54.8)	202 (55.2)
Men	160 (45.2)	164 (44.8)
Age, mean (SD), y	40.4 (9.3)	40.3 (8.9)
Religion		
Christian	353 (99.7)	363 (99.2)
Muslim	1 (0.3)	0
Other	0	3 (0.8)
Marital status		
Single	6 (1.7)	12 (3.3)
Married	251 (70.9)	271 (74.0)
Widowed	86 (24.3)	75 (20.5)
Divorced	3 (0.8)	3 (0.8)
Separated	8 (2.3)	5 (1.4)
No. of persons in household, mean (SD)	6.1 (2.7)	6.5 (2.6)
Wealth index quintile		
Lowest	80 (22.8)	62 (17.3)
Second	59 (16.8)	83 (23.1)
Third	67 (19.1)	76 (21.2)
Fourth	71 (20.2)	70 (19.5)
Highest	74 (21.1)	68 (18.9)
Depression score^b^		
Mean (SD)	1.5 (0.5)	1.7 (0.5)
Median (IQR)	1.4 (1.2-1.8)	1.7 (1.3-2.0)
Screening positive for depression		
No	248 (70.1)	196 (53.7)
Yes	106 (29.9)	169 (46.3)
BMI, median (IQR)	21.5 (19.7-23.7)	21.5 (20.0-23.8)
BMI categories		
Underweight	45 (12.7)	41 (11.3)
Normal	247 (70.0)	255 (70.4)
Overweight	44 (12.5)	49 (13.5)
Obese	17 (4.8)	17 (4.7)
Food security score, median (IQR)^c^	20.0 (17.0-24.0)	22.0 (20.0-25.0)
Categorical food security score		
Mild insecurity	2 (0.6)	1 (0.3)
Moderate insecurity	77 (21.8)	72 (19.7)
Severe insecurity	275 (77.7)	293 (80.1)
Viral load, detection limit ≤1000 copies/mL		
Undetectable	314 (89.0)	333 (91.0)
Detectable	39 (11.0)	33 (9.0)
Viral load, detection limit ≤200 copies/mL		
Undetectable	291 (82.4)	314 (85.8)
Detectable	62 (17.6)	52 (14.2)
Time since ART initiation, median (IQR), y	4.9 (2.6-6.9)	5.1 (2.7-7.2)
ART adherence, %		
95-100	331 (94.8)	344 (95.0)
75-94	17 (4.9)	17 (4.7)
<75	1 (0.3)	1 (0.3)

Abbreviations: ART, antiretroviral therapy; BMI, body mass index (calculated as weight in kilograms divided by height in meters squared).

^a^ Unless otherwise indicated, data are expressed as No. (%) of participants. Owing to missing data in some categories, denominators may be less than numbers in column headings. Percentages have been rounded and may not total 100.

^b^ Measured using the Hopkins Symptom Checklist for Depression^20^; values of 1.75 or greater were consistent with symptoms of depression.

^c^ Measured using the Household Food Insecurity Access Scale.^19^ Scores range from 0 to 27, with higher scores indicating greater insecurity.

### Baseline Data

At baseline, 149 participants (20.7%) had moderate food insecurity and 568 (78.9%) had severe food insecurity. Both groups had a median BMI of 21.5 (IQR, 19.7-23.8). Sociodemographic characteristics were generally balanced between both groups (Table 1). More intervention (169 of 365 [46.3%]) than control (106 of 354 [29.9%]) participants had positive screen findings for depressive symptoms at baseline. Median CD4 count was 542 (IQR, 395-708) cells/mm^3^ in the control group and 585 (IQR, 407-773) cells/mm^3^ in the intervention group.

#### Primary Outcome: Viral Suppression

**Figure 3.**
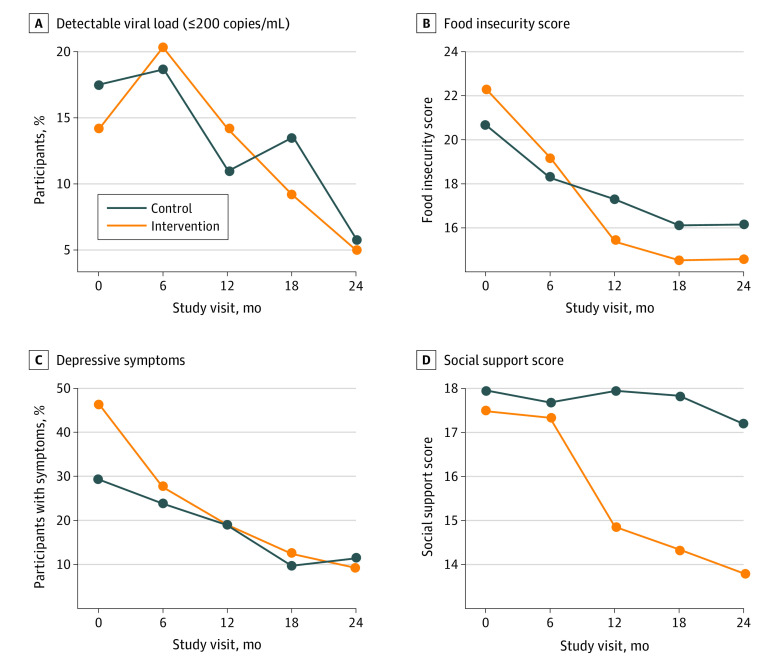
Selected Outcomes by Study Group and Visit Food insecurity scores range from 0 to 27, with higher scores indicating greater food insecurity. Social support scores range from 9 to 36, with higher scores indicating less social support.

In the intention-to-treat analysis, using a detection limit of 200 copies/mL or less, viral suppression in the intervention group increased from 314 of 366 participants (85.8%) at baseline to 327 of 344 (95.1%) at the 24-month visit compared with 291 of 353 (82.4%) at baseline to 314 of 333 (94.3%) at the 24-month visit in the control group (Table 2 and Figure 3A). Although the percentage of participants with viral suppression increased in both groups to approximately 95%, no difference was found between groups in the intention-to-treat (*P* = .86) or per-protocol (*P* = .85) analyses.

**Table 2.** Main Outcomes

Outcome	Study group^a^				Trend per 24 mo^b^		Difference in trend (95% CI)^c^	*P* value
	Control (n = 354)		Intervention (n = 366)		Control	Intervention		
	Visit 1	Visit 5	Visit 1	Visit 5				
HIV								
Undetectable viral load (≤200 copies/mL)	291/353 (82.4)	314/333 (94.3)	314/366 (85.8)	327/344 (95.1)	1.65	1.57	−0.08 (−1.0 to 0.84)	.86
CD4 (≤500 cells)	152/354 (42.9)	129/333 (38.7)	143/366 (39.1)	128/344 (37.2)	−0.38	−0.06	0.32 (−0.28 to 0.92)	.29
Hospitalized in the past 6 mo	20/353 (5.7)	18/333 (5.4)	34/366 (9.3)	21/344 (6.1)	−0.05	−0.39	−0.34 (−1.22 to 0.53)	.44
AIDS-defining condition	17/354 (4.8)	2/333 (0.6)	18/366 (4.9)	4/344 (1.2)	−2.13	−1.28	0.84 (−0.79 to 2.47)	.31
Food insecurity								
Food insecurity score, median (IQR)^d^	20.0 (17.0 to 24.0)	15.0 (14.0 to 19.0)	22.0 (20.0 to 25.0)	14.0 (11.0 to 18.0)	−4.51	−8.05	−3.54 (−4.16 to −2.92)	<.001
Food insecurity category								
Food secure	0	17/331 (5.1)	0	54/342 (15.8)	NA	NA	NA	NA
Mild insecurity	2/354 (0.6)	26/331 (7.9)	1/366 (0.3)	72/342 (21.1)	NA	NA	NA	NA
Moderate insecurity	77/354 (21.8)	215/331 (65.0)	72/366 (19.7)	106/342 (31.0)	NA	NA	NA	NA
Severe insecurity	275/354 (77.7)	73/331 (22.1)	293/366 (80.1)	110/342 (32.2)	NA	NA	NA	NA
Nutritional status								
BMI, median (IQR)	21.5 (19.7 to 23.7)	21.8 (19.0 to 24.5)	21.5 (20.0 to 23.8)	22.1 (20.2 to 24.6)	0.57	0.36	−0.21 (−0.39 to −0.04)	.02
Behavioral pathway								
Missed scheduled HIV visit in the past 6 mo	107/354 (30.2)	20/333 (6.0)	141/366 (38.5)	60/344 (17.4)	−2.05	−1.18	0.85 (0.24 to 1.49)	.007
ART adherence (self-report), median (IQR)^e^	100 (98.3 to 100)	100 (100 to 100)	100 (98.3 to 100)	100 (100 to 100)	0.54	0.55	0.01 (−0.82 to 0.84)	.98
ART adherence (self-report), %								
95-100	331/349 (94.8)	322/331 (97.3)	344/362 (95.0)	331/338 (97.9)	NA	NA	NA	NA
75-94	17/349 (4.9)	8/331 (2.4)	17/362 (4.7)	6/338 (1.8)	NA	NA	NA	NA
<75	1/349 (0.3)	1/331 (0.3)	1/362 (0.3)	1/338 (0.3)	NA	NA	NA	NA
Mental health								
Mental health score of MOS HIV, median (IQR)^f^	64.5 (50.5 to 74.9)	80.4 (71.8 to 84.8)	59.1 (45.3 to 69.8)	76.0 (59.6 to 84.4)	13.50	13.96	0.46 (−1.78 to 2.70)	.69
Physical health score of MOS HIV, median (IQR)^f^	85.8 (75.1 to 88.9)	86.2 (80.5 to 87.6)	83.5 (74.2 to 88.0)	86.0 (81.6 to 87.3)	1.11	2.50	1.39 (−1.01 to 3.78)	.26
Depression score, median (IQR)^g^	1.4 (1.2 to 1.8)	1.2 (1.0 to 1.4)	1.7 (1.3 to 2.0)	1.1 (1.0 to 1.3)	−0.26	−0.45	−0.19 (−0.34 to −0.04)	.01
Probable depression	106/354 (29.9)	41/333 (12.3)	169/365 (46.3)	36/344 (10.5)	−1.97	−2.80	−0.83 (−1.45 to −0.20)	.001
Social support score, median (IQR)^h^	18.0 (14.0 to 21.0)	18.0 (13.0 to 21.0)	17.0 (14.0 to 21.0)	13.0 (11.0 to 16.0)	−0.50	−4.13	−3.63 (−4.30 to −2.95)	<.001
Empowerment								
Self-confidence score, median (IQR)^i^	5.0 (4.0 to 6.0)	4.0 (4.0 to 6.0)	5.0 (4.0 to 7.0)	4.0 (4.0 to 6.0)	−0.37	−0.74	−0.37 (−0.59 to −0.15)	.001

Abbreviations: ART, antiretroviral therapy; BMI, body mass index (calculated as weight in kilograms divided by height in meters squared); MOS, Medical Outcomes Study; NA, not applicable.

^a^ Unless otherwise indicated, data are expressed as No./total No. (%) of participants. Percentages have been rounded and may not total 100.

^b^ Difference-in-differences estimates for all continuous outcomes were obtained using multilevel linear regression and are in units of the outcome.

^c^ Difference-in-differences estimates for undetectable viral load were calculated between visit 1 and visit 5 only (approximately 24 months). Difference-in-differences estimates for all binary outcomes were obtained using multilevel logistic regression and are in units of log odds.

^d^ Measured using the Household Food Insecurity Access Scale.^19^ Scores range from 0 to 27, with higher scores indicating greater insecurity.

^e^ Measured as proportion of pills taken compared with expected number of pills prescribed.

^f^ Scores range from 0 to 100, with higher scores indicating higher quality of life.

^g^ Measured using the Hopkins Symptom Checklist for Depression^20^; values of 1.75 or greater were consistent with symptoms of depression.

^h^ Scores range from 9 to 36, with higher scores indicating less social support.

^i^ Scores range from 0 to 9, with higher scores indicating less self-confidence.

In the evaluation of programmatic and clinic-based factors that could affect viral suppression at the cluster level, the viral load preparedness assessment score and rollout of dolutegravir were similar among cluster pairs (eTables 1 and 2 in Supplement 2). Dolutegravir, which was introduced in Kenya as first-line ART during the trial,^25^ was of interest because it is a more potent ART with a higher barrier to resistance than efavirenz.

In terms of other clinical indicators, the proportion of participants with a CD4 count of no greater than 500 cells/mm^3^ remained stable during follow-up (295 of 720 [41.0%] at visit 1 and 257 of 677 [38.0%] at visit 5), with no difference between groups (*P* = .29). The proportion hospitalized in the past 6 months was low in both groups at visit 1 (20 of 353 [5.7%] in the control group vs 34 of 366 [9.3%] in the intervention group), along with the proportion with an AIDS-defining condition (17 of 354 [4.8%] in the control group vs 18 of 366 [4.9%] in the intervention group), which declined during study follow-up. There were no differences between groups for either outcome (*P* = .44 and *P* = .31, respectively).

#### Food Insecurity

Food insecurity decreased more in the intervention group than in the control group (difference in linear trend, −3.54 [95% CI, −4.16 to −2.92] points; *P* < .001) during the 24-month follow-up (Figure 3B). At the end of follow-up, fewer participants in the intervention group (216 of 342 [63.2%]) than in the control group (288 of 331 [87.0%]) had moderate to severe food insecurity (*P* < .001).

#### Nutrition Pathway

Both study groups had small increases in median BMI, but the increase was slightly less in the intervention compared with the control group (difference in linear trends in BMI during the 24-month follow-up period, −0.21 [95% CI, −0.39 to −0.04]; *P* = .02). There was no difference in linear trends between the intervention and control groups in proportion with underweight (0.0024 [95% CI, −0.0300 to 0.0348]; *P* = .88) or with overweight or obesity (−0.0199 [95% CI, −0.0522 to 0.0125]; *P* = .23).

#### Behavioral Pathway

The proportion of participants who missed a scheduled HIV clinic visit in the past 6 months declined in both groups during follow-up, with a greater decline among participants in the control group compared with the intervention group (20 of 333 [6.0%] vs 60 of 344 [17.4%]; *P* = .007). At the 24-month visit among the 20 persons in the control group reporting a missed visit in the past 6 months, the most common reasons were traveling (n = 8), forgetting (n = 5), and illness (n = 3). Similarly, among the 60 persons in the intervention group reporting a missed visit in the past 6 months, the most common reasons were traveling (n = 24), other (n = 11), forgetting (n = 10), and illness (n = 5). Median self-reported 30-day ART adherence (measured as proportion of pills taken compared with expected number of pills prescribed) was high at baseline in both study groups (100 [IQR, 98.3-100] in the control group vs 100 [IQR, 98.3-100] in the intervention group) and did not change throughout the study period irrespective of study group (*P* = .98).

#### Mental Health Pathway

Although both the intervention and control groups experienced downward trends in depressive symptoms during the study period, the difference in trends (−0.19 [95% CI, −0.34 to −0.04] during the 24-month follow-up period; *P* = .01) indicated that the decline was greater in the intervention group compared with the control group. The prevalence of participants with probable depression declined in both groups during follow-up, with a greater decline in the intervention group (169 of 365 [46.3%] to 36 of 344 [10.5%]) than in the control group (106 of 354 [29.9%] to 41 of 333 [12.3%]; difference in trends, −0.83 [95% CI, 1.45 to −0.20]; *P* = .001) (Figure 3C). Similar to the depression outcomes, participants in the intervention group had a greater improvement in social support score than those in the control group (difference in trends, −3.63 [95% CI, −4.30 to −2.95]; *P* < .001) (Table 2 and Figure 3D).

#### Empowerment Pathway

The self-confidence measure improved in both groups, with a lower score indicating higher self-confidence. Participants in the intervention group experienced a larger change on the 5-point confidence scale during the 24-month study period than participants in the control group (difference in trends, −0.37 [95% CI, −0.59 to −0.15] points; *P* = .001) (Table 2).

#### Influence of Sex on Outcomes

Stratified by sex, there was a trend toward greater HIV viral suppression among men in the intervention group compared with the control group (difference in trend, 0.99 [95% CI, −0.22 to 2.20] points; *P* = .11), but not among women (difference in trend, −0.35 [95% CI, −1.41 to 0.71] points; *P* = .52) (eTables 3 and 4 in Supplement 2). Food insecurity improved for both sexes in the intervention group compared with the control group during the 24-month follow-up. The small increase in BMI for participants in the control group compared with the intervention group was limited to women (−0.45 [95% CI, −0.71 to −0.19]; *P* = .001), with no difference found among men (difference in trend, 0.09 [95% CI, −0.13 to 0.31]; *P* = .44). Other outcomes by study group were similar among men and women except impact on self-confidence, which was stronger among men (difference in trend, −0.63 [95% CI, −0.95 to −0.32]; *P* < .001), and the effect on depression, which was stronger among women (difference in trend, −0.25 [95% CI, −0.47 to −0.04]; *P* = .02). Additionally, men had improved physical health status scores on the Medical Outcomes Study HIV Health Survey (difference in trend, 4.71 [95% CI, 1.72-7.70]; *P* = .02) (eTables 3 and 4 in Supplement 2).

#### Potential Harms

During the trial, 5 participants in the intervention group and 4 participants in the control group died. No deaths were associated with study participation.

## Discussion

In this cluster RCT, we found that the *Shamba Maisha* intervention led to improvements in food security, mental health, self-confidence, and social support but not viral suppression, ART adherence, CD4 cell counts, or BMI for adult PLHIV receiving ART in rural Kenya. These findings support the potential for agricultural and livelihood interventions to address important underlying determinants of poor physical and mental health outcomes among PLHIV.

Change in viral suppression from baseline did not differ between study groups after 24 months of follow-up. Although our trial was adequately powered, the higher-than-expected rate of viral suppression in both study groups, which approached the UNAIDS 2030 goal for viral suppression of at least 95%,^26^ likely undermined our ability to achieve differences in this outcome. Among men, there was a trend toward greater viral suppression in the intervention group compared with the control group. The high rates of viral suppression in both groups occurred in the context of major structural changes to HIV treatment programs in Kenya during the time of our study, including implementation of wide-reaching test and treatment programs and the replacement of efavirenz with dolutegravir-based ART during the final 6 to 12 months of the trial. Although these programmatic interventions may have made it more difficult to detect impacts of the intervention on our primary end point, it is not atypical for clinical rollout of interventions to occur in the setting of multiple other interventions. Interestingly, these external program components were not in place during the pilot study conducted in 2012 and 2013, which found strong and significant differences in viral load suppression in intervention compared with control participants (odds ratio, 7.6 [95% CI, 2.2-26.8]; *P* = .02).^18^ Thus, agricultural livelihood interventions such as *Shamba Maisha* may be more effective in improving HIV outcomes among PLHIV in regions with less effective HIV treatment programs^27^ and within subpopulations in sub-Saharan Africa who have lower rates of viral suppression, such as pregnant and postpartum women, adolescents, and young adults.^28,29,30^

Participants in the intervention group achieved a greater improvement in food security compared with the control group. Food insecurity is a critical determinant of HIV acquisition, tuberculosis treatment, and development of noncommunicable diseases such as hypertension, type 2 diabetes, and heart disease; it is also a key predictive factor associated with mental health outcomes such as depression, anxiety, and posttraumatic stress disorder.^31,32,33^ These consequences of food insecurity occur through both nutritional (eg, underweight or overweight) and nonnutritional (eg, nonadherence to treatment and care, inflammation) mechanisms. Given the bidirectional associations between food insecurity and HIV, mental health, and chronic diseases, future studies should consider testing the effectiveness of agriculture and livelihood interventions such as *Shamba Maisha* on concurrent health conditions. Identifying synergies among health conditions to design cost-effective interventions could maximize public health benefits across multiple health outcomes and within targeted populations.^34^

The *Shamba Maisha* cluster RCT is among the first studies to demonstrate that a livelihood intervention can reduce depression. Livelihood interventions that address food insecurity have the potential to improve mental health through multiple mechanisms, including improved food security and income, increased physical activity and productive labor, an improved sense of self, and contribution to one’s community.^35,36^ Improving mental health is a major global health priority; between 2011 and 2030, mental illness is projected to cost the global economy $16 trillion US in lost economic output—more than cancer, diabetes, and respiratory diseases combined.^37^ Although interpersonal psychotherapy delivered by nonspecialists and medication can lead to improvements in mental health outcomes,^35^ these modalities do not address the upstream determinants of poor mental health, such as food insecurity and poverty. As such, agriculture and livelihood interventions can play an important role in improving mental health outcomes.

Although other livelihood interventions have demonstrated efficacy to improve quality of life, psychological well-being, and self-efficacy,^38^ *Shamba Maisha* is among the first to demonstrate improvements in self-confidence and social support among PLHIV.^39,40^ These independent findings may help explain the reduction in depression witnessed among participants in the intervention group.^41^

### Strengths and Limitations

This trial has many strengths, including the cluster randomized design, use of validated measures and objective clinical indicators, and a 2-year retention rate of nearly 95% in both groups. Although the randomization of the 16 facilities resulted in some small differences in outcome measures at baseline, these differences were mitigated because each participant served as their own control. Last, extraneous factors such as record droughts and floods that in some cases led to crop destruction may have affected the impact of the intervention.

This trial also has some limitations. We limited enrollment to adults living with HIV and receiving ART in rural Kenya who had food insecurity and who had some farming experience and access to land and water for irrigation, which may limit generalizability, particularly in the setting of worsening climate change. Importantly, 85% of persons in rural Kenya depend on agriculture for their living, and the density of farms is highest in the areas with easiest access to water, suggesting that a sizeable proportion of the population could benefit from similar agricultural interventions. Although our findings should not necessarily be extrapolated to other regions and populations, other agricultural livelihood interventions have demonstrated impacts in other settings on outcomes such as household food insecurity, agricultural practices, women’s empowerment, and women’s well-being.^42,43,44,45^ Owing to changes in banking regulations in Kenya that occurred just before initiating our study, delays in loan disbursal prevented acquisition of agricultural assets in a timely manner, which may have prevented some participants from maximally benefiting from the intervention. Future livelihood interventions should also consider other approaches such as granting commodities that may even be less costly to scale up in the long term.

High-burden HIV areas tend to be heavily concentrated in regions of the world that are highly vulnerable to the impact of climate change; agriculture and livelihood interventions resilient to rainfall deviations, including droughts and floods, could therefore be well positioned to improve HIV-related and other health challenges stemming from food and water insecurity in the long term.^46^ *Shamba Maisha* is unique as an agriculture and livelihood intervention for PLHIV in that we incorporated into its design a drought-focused element (a human-powered irrigation pump) and training on sustainable farming techniques, including practicing regenerative agriculture. Although participants in our study were challenged by historic flood and drought conditions in western Kenya, the intervention led to demonstrable health and other benefits, suggesting that the intervention helps farmers to adapt to climate change.

## Conclusions

In this cluster RCT, because viral suppression approached the UNAIDS goal of at least 95% in both study groups in the setting of widespread test and treatment policies launched during the study period,^47,48^ it was not possible to detect additional effects of the multisectoral agricultural intervention on HIV clinical indicators. The intervention reduced food insecurity and depressive symptoms and improved self-confidence and social support among PLHIV. Interventions that improve livelihoods should focus on alleviating these constraining underpinnings while aiming to directly address multiple poor health conditions that may be syndemic.

## Notes

Supplement 1.
Trial Protocol

Supplement 2. **eMethods.** Additional Data Collection and Analysis **eTable 1.** Viral Load Readiness Assessment Score Among 8 Pairs of Intervention and Control Facilities **eTable 2.** Proportion of Participants With an HIV RNA Viral Load <200 Copies/mL at Baseline and Using Dolutegravir-Based Antiretroviral Therapy at the 24-Month Follow-up Visit Among the 8 Pairs of Intervention and Control Facilities **eTable 3.** Main Outcomes Among Male Participants **eTable 4.** Main Outcomes Among Female Participants

Supplement 3.
Data Sharing Statement
