# Supplementary material for: Effect of a Multisectoral Agricultural Intervention on HIV Health Outcomes Among Adults in Kenya: A Cluster Randomized Clinical Trial
Source: JAMA Netw Open. 2022 Dec 12;5(12):e2246158. doi: 10.1001/jamanetworkopen.2022.46158 (PMC9856331; doi:10.1001/jamanetworkopen.2022.46158)
Supplement: Supplement 1. — Trial Protocol [file jamanetwopen-e2246158-s001.pdf]

Shamba Maisha Statistical Analysis Plan (project 1R01MH107330-01) June 10, 2020

Contents

Section 1: Administrative Information 3
1) Title and trial registration 3
2) SAP version number with dates 3
3) Protocol version 3
4) SAP revisions 3
5) Roles and responsibilities – names, affiliations, and roles of SAP contributors 3
6) Signatures 3
Section 2: Introduction 3
7) Background and rationale 3
8) Objectives 4
Section 3: Study Methods 4
9) Trial design 4
10) Randomization 4
11) Sample size 5
12) Framework 6
13) Statistical interim analyses and stopping guidance 6
14) Timing of final analyses 6
15) Timing of outcome assessments 6
Section 4: Statistical Principles 6
16) Level of statistical significance 6
17) Description and rationale for any adjustment for multiplicity 6
18) Confidence intervals 6
19) Adherence and protocol deviations 6
21) Analysis populations 8
a. Intent to treat 8
b. Modified intent to treat - 8
c. Per protocol: 8
i. The per protocol analysis will include all control participants 8
d. Complete case— 8
Section 5: Trial Population 8

22) Screening Data 8
23) Eligibility 8
24) Recruitment 9
25) Withdrawal/follow-up 9
26) Baseline patient characteristics 10
Section 6: Analysis........ 10
27) Outcome definitions 10
28) Analysis methods 15
29) Missing data 17
30) Additional analyses 17
31) Harms 17
32) Statistical software –........ 18
33) References 18
34) Appendix: 22
a. Appendix 1: Description of the intervention components 22

Section 1: Administrative Information

1) **Title and trial registration**
    a. *Shamba Maisha*: Agricultural intervention for food security and HIV health outcomes in Kenya
    b. Trial registration number: NCT02815579

2) **SAP version number with dates**
    a. V1 – 10 June 2020

3) **Protocol version**

    This SAP references Shamba Maisha Protocol Version 9.0, last updated March 27, 2019

4) **SAP revisions**
    a. SAP revision history
    b. Justification for each SAP revision
    c. Timing of SAP revisions in relation to interim analyses, etc.

5) **Roles and responsibilities – names, affiliations, and roles of SAP contributors**
    a. Edward A. Frongillo, Jr., PhD—Senior statistician for Shamba Maisha study. University of South Carolina, Columbia.
    b. Adrienne Rain Mocello, MPH—Data manager and analyst for Shamba Maisha study. University of California, San Francisco.
    c. Lila A. Sheira, MPH—Data manager and analyst for Shamba Maisha study. University of California, San Francisco.
    d. Rachel Burger, MPH—Shamba Maisha Study Director. University of California, San Francisco.

6) **Signatures**
    a. Multiple-PIs
    b. Senior statistician

Section 2: Introduction

7) **Background and rationale**

    Despite major advances in care and treatment for those living with HIV, morbidity and mortality among people living with HIV/AIDS (PLHIV) remain unacceptably high in sub-Saharan Africa (SSA). Food insecurity and poverty contribute to higher morbidity and mortality among PLHIV, and there has been increasing international recognition of the need to address these factors for a successful global response to the HIV epidemic. Yet, to date there have been few studies to systematically evaluate the impact and cost-effectiveness of promising food security interventions on health outcomes among PLHIV. To address these gaps, together with KickStart, a non-governmental organization based in SSA, we developed a multisectoral intervention in Nyanza Region, Kenya that includes: a) a loan (~$175) for purchasing agricultural implements and commodities; b) agricultural implements to be purchased with the loan including a human-powered water pump, seeds, fertilizers and pesticides; and c) education in financial management and sustainable farming practices. We successfully completed a pilot intervention trial that showed that the intervention was feasible, acceptable and may improve HIV-related

health. We conducted a cluster randomized controlled trial (RCT) of this intervention with the following specific aims:

8) Objectives

Aim 1: To determine the impact of a multisectoral agricultural intervention among HIV-infected farmers on ART on HIV clinical outcomes. We hypothesize that the intervention will lead to improved viral load suppression (primary outcome) and decreased HIV-related morbidity in the intervention arm compared to the control arm.

Aim 2: To understand the pathways through which the multisectoral intervention may improve HIV health outcomes. Using our theoretical model, we hypothesize that the intervention will improve food security and household wealth, which in turn will contribute to improved outcomes through nutritional (improved diet quality, nutritional status), mental health (less depression, improved empowerment), and behavioral (improved ART adherence, and retention in care) pathways.

Aim 3: To determine the cost-effectiveness of the intervention and obtain the information necessary to inform scale-up in Kenya and similar settings in SSA. We hypothesize that the intervention will be cost-effective, and that we will be able to translate lessons learned into successful scale up.

Section 3: Study Methods

9) Trial design

We conducted a matched pair cluster randomized controlled trial (RCT) of a multisectoral agricultural intervention among HIV-infected farmers on ART to determine the impact of the intervention on HIV viral load suppression (primary outcome), and additional health outcomes including changes in CD4 cell counts, WHO Stage III/IV disease, hospitalizations, physical health status and mortality (secondary outcomes). We also set out to determine the impact of this intervention on mediating outcomes in the hypothesized causal framework. Proximal mediators included food security and household economic indicators. We also assessed nutritional (diet quality, anthropometry), behavioral (ART adherence, engagement and retention in care), mental health (depression, mental health status), and empowerment (gender roles, relationship power, self-confidence, self-efficacy and household decision-making) mediators as per our causal framework. Alongside the trial, we conducted a cost-effectiveness analysis and a process evaluation, which included interviews with study participants, staff and various stakeholders to understand strengths and pitfalls of the intervention and translate lessons learned to guide a possible scale-up of the intervention in similar settings in East Africa.

10) Randomization

Eight matched pairs of facilities were selected for inclusion in the study in a 1:1 ratio to the intervention and control arms. Pair matching helps protect study credibility and validity with a limited numbers of clusters, and also can substantially improve power.[1,2] With newer matching algorithms/schemes, we can get close matches with multiple variables predictive of the outcome.[3,4,5,6] Therefore, we matched facilities on the following criteria: 1) size of facility (district, sub-district hospital, health center, dispensary) 2) geography defined by sub-county, 3) primary source of water for irrigation (lake, river, shallow wells), and 4) access to markets. We selected matched pairs that limit the chance of contamination between intervention and control health facilities based on geographic proximity and ethnographic mapping. Based on our pilot work, the minimum requirement for health facility inclusion in the study were: Ministry of

Health facility that serves a minimum of 350 patients on ART, proximity to permanent water source/s (river, lake, and streams), suitable soil for farming, proximity to markets, and where farming is one of the a key economic activities in the community (i.e.: 50% of the population is involved in agriculture as the primary means of income, which applied to most of the Ministry of Health facilities in Nyanza Region). Randomization, based on a computer-generated assignment, occurred after the eight matched pairs have been selected.

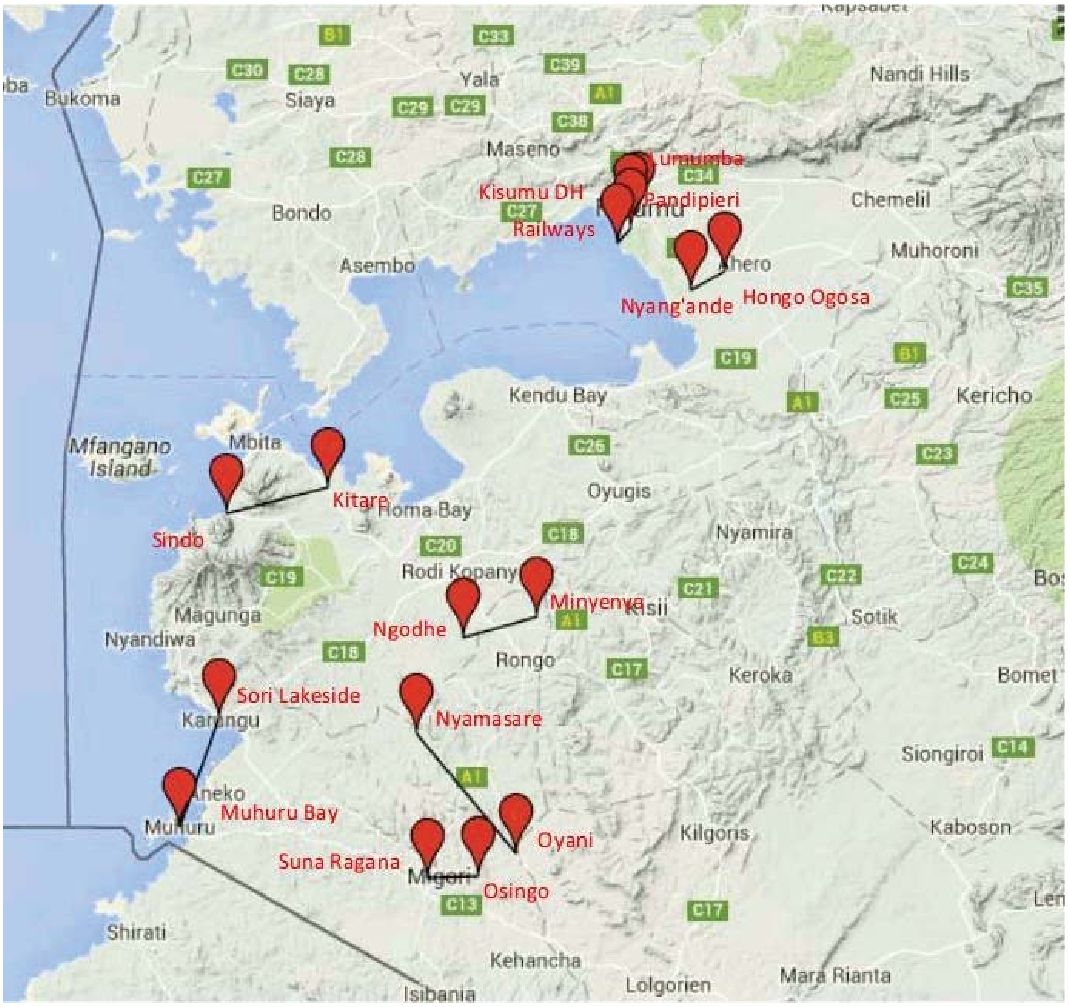

11) Sample size

Data from the Shamba Maisha pilot R34 intervention study were used to estimate the sample size needed for this study for the outcomes of changes from month 0 to month 24 in the food insecurity score (key mediating variable), CD4 count, and viral load suppression (key outcome variables). We assumed that the standard deviations (SD) seen in the current study would be similar to that in the pilot study because the two studies drew from a similarly geographically dispersed population.[7] To be conservative, we assumed a coefficient of variation due to clustering of 0.150, ignoring the matched pairs.[8] Retention in the pilot study was 98%; to be conservative, retention in the current study was assumed to be 90%. For two-sided testing at α=0.05 in a longitudinal analysis, a sample of 8 health facilities per arm with up to 44 enrolled participants per health facility (total enrollment of up to 352 per arm) will provide power of 80% for an important clinical difference of 0.138 between the intervention and control arms in the proportion becoming virally suppressed from month 0 to month 24 (primary outcome).[8] We over-enrolled the

number of intervention participants by up to 55 per facility (compared to 44 in the control arm) to account for participants that we anticipated might not be able to save the down payment. That is, assuming as in our pilot study that 0.150 of the sample in the control arm becomes suppressed from baseline to month 24, there is 80% power to detect differences such that the proportion becoming suppressed from baseline to month 24 in the intervention arm is 0.288 or greater. With regard to two secondary outcomes, the within-arm SD for changes in food insecurity score and CD4 count were estimated from the pilot study as 2.95 and 208.9 cells/mm3, respectively. The sample size provided power of 80% for differences of 1.2 for food insecurity (HFAIS score) and ≥57 cells/mm3 for CD4 count.

12) **Framework**

We will use the superiority hypothesis testing framework, testing whether exposure to the intervention results in better outcomes than exposure to the control standard of care. Comparisons will be presented as differences between arms in changes in outcomes from baseline to endline.

13) **Statistical interim analyses and stopping guidance**
    a. Information in interim analyses specifying what interim analyses will be carried out and listing time points
        i. None planned or performed
    b. Any planned adjustment of the significance level due to interim analysis
        i. No
    c. Details of guidelines for stopping the trial early
        i. None

14) **Timing of final analyses**

Baseline analyses began in May of 2019. Final analyses will begin in mid-2020 upon completion of all field data collection in December 2019), and freezing of the database in the second quarter of 2020.

15) **Timing of outcome assessments**

Research staff administered surveys to both arms at baseline and at 6, 12, 18, and 24 months. Data were collected within a window period around each data collection time point of +/- 2 months.

<u>Section 4: Statistical Principles</u>

16) **Level of statistical significance**

No significance testing will be conducted unless required by a journal. We will report 95% confidence intervals and exact p-values (or p<0.001).

17) **Description and rationale for any adjustment for multiplicity** and, if so, detailing how the type I error is to be controlled.

Our primary outcomes were established in our protocol, and thus no adjustments will be made for multiplicity.

18) **Confidence intervals**

95% confidence intervals will be reported alongside exact p-values.

19) **Adherence and protocol deviations**
    a. Definition of exposure to the intervention and how this is assessed including extent of exposure:
        i. Receipt of pump and agricultural inputs (seeds, etc.) AND

    ii. Receipt of at least six of eight essential agriculture training sessions and one of two financial training sessions

b. Description of how adherence to the intervention will be presented - consort diagram and brief description in the narrative
c. Definition of protocol deviations for the trial

20) **Protocol deviation** is defined as any failure to achieve full exposure to the defined procedures or treatment plans outlined in the study protocol version previously approved by the IRB except if intended to eliminate a hazard to the study participant or protect the wellbeing or life of the study participant in an emergency. The noncompliance may be either on the part of the investigator or the study site staff and may result in significant added risk to the study subject. As a result of deviations, corrective and preventive actions are developed by the site and implemented promptly. Protocol deviations are summarized below

    a. We saw 19 participants for study visits outside of the initial protocol specified visit window (+/- 1 month). All efforts were made to reach the participants on time but the participants were not available. Study activities had to be stopped from mid-April 2017 to mid-June 2017 because there was a lapse in the annual KEMRI renewal approval. No study activities were carried out during the elections period (7 August, 2017- 18 August, 2017). The participants were not able to be located within the window and therefore they were seen outside of the visit window. As a result, we updated the standard operating procedure (SOP) that addresses follow up and retention of participants. The SOP outlines that the research assistants will make calls and conduct home visits in case a participant misses his or her appointment date to ensure that the participants are seen with the appointment window period. We also subsequently submitted an IRB modification to expand the visit window to be +/- 2 months to avoid subsequent protocol deviations.
    b. An electronic and printed copy of the renewal application was submitted to KEMRI Scientific and Ethical Review Unit (SERU) on 23 February 2017 in advance of the SERU deadline 3 March 2017. The printed copies did not reach the appropriate office and the study team was not notified. SERU requires both printed and electronic submissions and as a result, our study approval lapsed at KEMRI on 14 April 2017. We halted recruitment of new participants and follow-up data collection for existing participants until IRB approval was granted. Additional follow-up has been done to confirm receipt of both printed and electronic submissions on the date of submission. Additional follow-up in advance of expiration date is also done to avoid any lapses in approval.
    c. Early in the trial we discovered that one of our research assistants had not conducted the required home visits for 22 participants. After extensive investigation and disciplinary action, the RA was dismissed from the study, and the 22 participants had their home and clinic visits repeated. All 21 Research Assistants were retrained on the ethical conducts in research. An automatic start and end date and time was added on all forms for future verification.
    d. One participant was incorrectly enrolled in the study (04-44). The individual did not have permanent surface water available next to her farm based on the home screening report. The participant was informed by the study coordinator that she had been erroneously enrolled and would be withdrawn from the study. We screened three more participants in the facility where the error occurred for replacement. All the Research Assistants were provided a refresher training on the eligibility criteria.

21) **Analysis populations** – definition of analysis populations, e.g. intention to treat, per protocol, complete case, safety

a. Intent to treat - The primary analysis will be intent to treat (ITT). The ITT analysis will include all participants in both arms who were enrolled and completed all baseline assessments. We will exclude: 16 participants who were unenrolled due to lack of payment of the down payment. This requirement was removed from the study protocol after these individuals were unenrolled. The following participants are considered immediate withdrawals and will also be excluded from the ITT analysis: 4 participants who withdrew consent (3 intervention participants before attending any trainings and 1 control participant immediately after enrollment); 2 intervention participants moved out of the study area (before receiving any training); 1 intervention participant was hospitalized immediately after enrolment and hence was not available to partake in the study; 1 intervention participant was uncomfortable using MEMS; 1 intervention participant withdrew per participant request; and 1 control participant who was found not to have met the study criteria.

b. Modified intent to treat - A modified intent to treat analysis will include all control participants in the ITT sample and all intervention participants who received the loan and at least one agricultural or financial training.

c. Per protocol - The per protocol analysis will include all control participants in the modified ITT sample and those intervention participants in the modified ITT sample who received six of the eight essential agricultural training sessions, one of the two financial trainings, a pump, and agricultural inputs. See appendix 1 for details about the trainings.

d. Complete case— We will not conduct complete case analyses as we will attempt to use all available data from all participants (see section 29: Missing Data).

Section 5: Trial Population

22) **Screening Data** – reporting of screening data to describe representativeness of trial sample by gender.

a. Participants were recruited through organized meetings held at each health facility, publicized through announcements at patient support group sessions. At each meeting, research staff presented study details and eligibility guidelines. Home visits were conducted among interested and potentially eligible individuals to verify that the participant has access to agricultural land and surface water. Using female recruiters and promotional material geared towards recruiting women, we enrolled at least 40% of participants at each health facility from each gender.

23) **Eligibility** – summary of eligibility criteria

At both intervention and control health facilities, we enrolled up to 55 persons currently enrolled in HIV care per health facility. Eligibility criteria were similar those in our pilot study:

1. HIV-infected adults
2. 18-60 years old
3. Currently receiving ART
4. Belong to a patient support group or demonstrate willingness to join one.
5. Have access to farming land and available surface water in the form of lakes, rivers, ponds and shallow wells.
6. Have evidence of moderate to severe food insecurity based on the Household Food Insecurity Access Scale (HFIAS), and/or malnutrition (BMI<18.5) based on medical records during the year preceding

recruitment.

7. Participants must also agree to save the down payment (no more than 2,000 KSH) required for the loan

Criteria for exclusion of subjects

- People who do not speak Dholuo, Swahili, or English
- Inadequate cognitive and/or hearing capacity to complete planned study procedures, at the discretion of the research assistant

24) **Recruitment** – information to be included in the CONSORT flow diagram
    a. Screening was conducted in two stages, during an initial clinical recruitment visit and at a follow-up home screening visit. During the clinical screening, potential participants were first consented for study screening and then checked for eligibility criteria 1 through 7. Participants who met all criteria then proceeded to the home screening, where their access to farming land and surface water was verified, as well as the location of their home within the predefined study area.
    b. After recruitment was completed at the first four sites, in response to the high proportion of viral suppression among enrolled participants, we changed our enrollment procedures to ensure that at least 40% of participants at each site had a detectable viral load or fair-to-poor adherence to ART or a missed visit greater than three days. To reach these targets, we conducted chart reviews prior to screening participants to identify individuals with:
        i. A detectable viral load (> 50 copies/ml) in the prior 12 months among patients on ART for ≥ 6 months
        ii. An ART treatment interruption or fair-to-poor ART adherence
        iii. A missed visit by more than 3 days in the prior 12 months
    c. Screening numbers
        i. 606 people were screened at 8 intervention sites
            1. 216 (36%) were not enrolled
        ii. 521 people were screened at 8 control sites
            1. 165 (32%) were not enrolled
    d. See CONSORT diagram for detail on reasons for ineligibility

25) **Withdrawal/follow-up**
    a. Level of withdrawal, e.g. from intervention and/or from follow-up
        i. Participants were withdrawn from the study due to failure to meet study criteria, unavailability immediately following enrollment (e.g. moved out of the area, hospitalized), and through immediate participant request (withdrawal of consent, discomfort with study procedures). Additionally, at the beginning of the study, 16 participants were withdrawn early by the investigative team due to failure to make the loan down payment (Version 5.0 of the protocol). This requirement was subsequently discontinued.
            1. 24 (6%) of enrolled intervention participants were withdrawn from the study
            2. 2 (1%) of enrolled control participants were withdrawn
        ii. LTFU occurred through migration out of the study area, imprisonment, and death. Intervention participants were also considered LTFU if they withdrew consent.

        1. At month 24, 75% of intervention participants were retained from month 0.
        2. At month 24, 80% of control participants were retained from month 0.
   b. Timing of withdrawal/LTFU data
      i. Intervention participants left the study prior to their first training they were considered withdrawn, otherwise they were considered LTFU.
      ii. Control participants could be withdrawn immediately after enrollment otherwise they were considered LTFU.
   c. Reasons and details of how withdrawal/LTFU data will be presented
      i. Withdrawal and LTFU data are presented in the CONSORT diagram, stratified by study arm and visit. These data are included to establish lack of bias in screening and retention between the two arms.

26) **Baseline patient characteristics**

We will evaluate several socio-demographic and clinical characteristics at baseline by which we will describe our study sample. These are outlined in Table 1 below and a shell table is provided in the appendix. We will stratify these characteristics by intervention and control arms and report median and the inter-quartile range for continuous variables and the N and percent for categorical and dichotomous variables. No significance testing or p-values will be conducted or reported per CONSORT guidelines.

**Table 1: Baseline characteristics of Shamba Maisha participants**	
Socio-demographic	Age, sex, household size, marital status, polygamous marriage or not, educational attainment
Economic welfare	Household food insecurity, household per-capita food expenditures, household wealth
Clinical	Number of years living with HIV, number of years on ART, current regimen, recent hospitalizations, history of opportunistic infections
Mental Health	Mental health summary score (from MOS-HIV), depression, alcohol abuse
Behavioral	Self-reported ART adherence levels, missed healthcare visits
Physical Health	Physical health summary score (from MOS-HIV)
Clinical outcomes	Viral load, CD4 cell count, recent hospitalizations

<u>Section 6: Analysis</u>

27) **Outcome definitions**
   a. Aim 1 Outcomes:
      i. <u>HIV RNA viral load</u>: The primary outcome for Aim 1 will be HIV RNA viral load suppression, which was assessed at each visit, i.e. every six months, among all study participants. Blood was collected during their laboratory visit and sent to a regional lab for analysis. Results were received in copies/mL of blood. In the case of a missed visit, participants could complete their blood draw within 2 months of their scheduled visit date. For analysis of the primary outcome, we will compare the proportion of participants virally suppressed, defined as ≤200 copies/mL at baseline compared to endline between the two study arms. As an exploratory analysis, we will also examine HIV RNA viral load continuously, using a natural log transformation to account for a non-normal distribution and using a secondary cut-offs of <1000.

ii. HIV related morbidity: As a secondary Aim 1 outcome, we collected or abstracted data on hospitalizations, opportunistic infections, hospitalizations, and HIV symptoms. From the data on opportunistic infections, we will develop the following variables, both of which will be analyzed dichotomously: WHO HIVAIDS clinical stage (stages I-IV) and presence of any AIDS-defining events. [9] From the data on hospitalizations, we will make two dichotomous variables: 1) any hospitalizations in the last six months and 2) number of times admitted to the hospital in the previous six months.

b. Aim 2 Outcomes: There are several intermediate outcomes. The primary intermediate outcomes are linear trends over all five visits in food security, depressive symptoms, ART adherence, frequency of food intake, self-confidence, and household agricultural income.

i. Food Security: We measured food security status using the Household Food Insecurity Access Scale (HFIAS). This scale has been validated in eight countries [10,11] and used successfully by our team in Kenya and rural Uganda. [12,13,14,15,16,17,18] The nine-item questionnaire covers domains of sufficiency, quality, access, and supply. The scores will be analyzed primarily as a continuous variable using a sum of all responses with a possible range of 9-27, with higher scores being indicative of less food security. We will examine the HFIAS score continuously as our primary analysis. As a secondary analysis, we will assess the HFIAS categorically, which will be assessed in two different ways: 1) using standard scoring algorithms that result in a participant having either food security, mild food insecurity, moderate food insecurity, or severe food insecurity; and 2) dichotomously, using the categorical variable to generate a new variable that is either food secure or any food insecurity.

ii. Food expenditures: We collected data on expenditures on all a comprehensive list of common food staples and items (which corresponds to the list of foods in the food frequency questionnaire). We will create a number of variables which corresponds to the per-capita expenditures per food group (eggs, fish, dairy, caffeine drinks, condiments, sweets, cooking fats, fruit, meat and poultry, grains, roots/tubers, vegetables, and legumes) as well as a variable that is the total per-capita food expenditures (the sum of all the aforementioned groups). This variable will be analyzed continuously. This expenditure variable quantifies the amount spent on food purchases and does not quantify amounts of food from home production or gifts; constructing a food consumption variable that includes home production and gifts requires developing and implementing a strategy to monetize the amount of production and gifts by identifying an appropriate price per unit in order to calculate the total per-capita value of food consumption.

iii. Frequency of food intake: Food frequency will be measured as the number of different foods or food groups consumed over a given period,[19] as used in the Kenya Demographic and Health Survey.[20] Food groups and their frequency of consumption will be computed following the method used in the pilot study, which generates the following food groups: eggs, fish, dairy, caffeine drinks, condiments, sweets, cooking fats, fruit, meat and poultry, grains, roots/tubers, vegetables, and legumes. We will then generate a continuous variable that is overall consumption in portions per day of all the

food groups combined and compare this in the intervention and control arms over the study period.

iv. Household wealth: We measured household wealth using the Demographic and Health Surveys wealth index, a composite measure of living standard. The wealth index is calculated using self-reported data on household assets, household materials such as walls, roof, and floor materials, water sources, and sanitation facilities. Following DHS scoring guidance, we used a principal components analysis to construct the wealth index. We then made two variables to assess household wealth: 1) a continuous score where a higher score indicates more wealth (primarily); and 2) a categorical score of wealth quintiles, where the first quintile indicates the lowest 20% and the fifth quintile indicated the highest 20% (secondary).

v. Nutritional status: We measured Body Mass Index (BMI) and Mid-Upper Arm Circumference (MUAC), commonly used to assess nutritional status.[21,22]

    1. BMI reflects protein and fat reserves[23] and will be examined as a continuous variable to assess trends in change of BMI. As a secondary analysis, we will use an established grading system of underweight, normal BMI, overweight, and obese.[24]
    2. For MUAC, we will use WHO sex-specific cut-offs of 22.0 cm for women and 23.0 cm for men with chronic energy deficiency.[25]

vi. Mental health:

    1. Mental health status was measured using the MOS-HIV, a tool for assessing health-related quality of life[26] that has been validated among HIV-infected populations in resource-limited settings.[27,28] We will use a two-factor confirmatory factor analysis to develop the mental and physical health summary scales, per standard scoring. The scores will be rescaled to have a range of 0-100, with higher scores being indicative of better health in each domain.
    2. Depression was screened using the Hopkins Symptom Checklist for Depression, a 15-item scale[29] which has been validated in sub-Saharan Africa.[30] The scale will be developed per standard scoring algorithms and analyzed both continuously, with higher row average scores indicative of more depressive symptoms (primary analysis), and categorically, with those with an average row score of 1.75 or greater screening positive for depressive symptoms (secondary analysis).
    3. Alcohol use: We used the AUDIT-C indicators. The AUDIT-C is a 3-item alcohol screen that can help identify persons who are hazardous drinkers or have active alcohol use disorders.[31] We will review the AUDIT-C score continuously stratified by sex, however given that cut-offs for hazardous drinking differ by sex, we will examine this dichotomously for main analyses. The cutoff for hazardous drinking on the AUDIT-C in males and females is a score of 4 and 3, respectively. Refer to section 28.a to see how non-standardized drinking quantities were handled.
    4. HIV-related stigma and disclosure: We will use the Internalized AIDS-Related Stigma Scale, which has been extensively validated in sub-Saharan African

settings.[32] The HIV-related stigma scale asks about internalized, anticipated, and enacted stigma, and three sub-scales for each domain can be developed based on the responses. The three sub-domains will be analyzed continuously, with higher scores being indicative of more stigma in each of the respective sub-domains. We asked about disclosure of HIV status to partners, family members, and others using questions adapted from our previous studies in SSA.[33,34,35]

vii. <u>Empowerment</u>:

1. Women's empowerment indicators were adapted from a large cluster-randomized trial of an intervention including: greater challenges to established gender roles, communication with relationship partners about sexual matters, measures of financial decision-making, measures of attitudes towards gender roles and gender-based violence, and experience of controlling behavior by relationship partner.[36] Specifically, there are three scales which we collected data on and plan to analyze:
    a. Self-confidence scale: To measure empowerment, we adapted three Likert-type items from the IMAGE Study around self-confidence and financial confidence.[37] The items ask about speaking in public, offering advice to a neighbor about children or farming, and independently raising money to feed one's family for a month. Responses comprise an index ranging from 1 (very confident) to 3 (not confident at all) and were also examined as a dichotomous measure with a cut-off of 4 or more indicating empowerment. We will examine the self-confidence scale as a continuous measure primarily, followed by the dichotomous cutoff as a secondary analysis approach.
    b. Sexual Relationship Power Scale (SRPS),[38] which conceptualizes sexual relationship power as a two-dimensional construct assessing degrees of relationship control and decision-making dominance. The SRPS has been used successfully in observational research conducted in the United States [maybe put original Pulerwitz piece here?], South Africa[39,40] and Uganda.[15] Responses are scaled and summed into an overall SRPS scale and into two subscales (relationship control and decision-making dominance). We will examine the overall scale and each of the sub-scales divided into tertiles, with higher scores indicating higher sexual relationship power and independent decision making, respectively.
    c. Gender role conflict scale: The gender role conflict scale is 22-items asked of male participants which encompasses four patterns of gender role conflict: 1) Success, Power, and Competition (SPC), 2) Restrictive Emotionality (RE), 3) Restrictive Sexual and Affectionate Behavior Between Men (RABBM), and 4) Conflict Between Work and Family Relations (CBWFR).[41] Responses are summed to make a continuous score with lower scores indicative of lower gender role conflict.
2. Intimate partner violence: We collected data at baseline and each subsequent visit on the perpetration or victimization of intimate partner violence among

males and females, respectively. The five questions asked about physical abuse, forcing one to leave home, and/or sexual abuse and trickery. We made two primary variables: 1) lifetime experience of IPV (reported any abuse at baseline) and 2) recent IPV (any IPV since the last visit vs. not). As a secondary analysis, we will look at three variables: emotional, physical, and sexual abuse (past 6 and 12 months).

viii. Antiretroviral therapy (ART) adherence

1. Self-report: We measured self-reported ART adherence in two ways. First, we used the visual analog scale,[42] which corresponds to the percentage of prescribed doses taken, and is correlated with unannounced pill count and MEMS.[43,44,45] Secondly, we will calculate a monthly percent adherence based on the number of doses a participant reports per day, and the number of self-reported missed doses over a month.
2. ART adherence: Participants received a Medical Electronic Monitoring System (MEMS) bottle to record bottle opening events providing a graphical printout of adherence. MEMS is one of the most extensively validated objective measures of ART adherence for use of studies in sub-Saharan Africa, is closely correlated with undetectable viral loads,[46] and has been found to be feasible and acceptable to patients in the Nyanza Region by our study team.[47] [45] We will analyze MEMS opening data continuously and dichotomously using an 80% cut-off for adherence. Additionally, we will assess treatment interruptions using the two methods. First, we will count the number of consecutive days without a device opening. A period of seven or more days without a device opening will be counted as a treatment interruption. Second, we will calculate a running average of adherence per participant per day as the average of the surrounding 9 days, starting at 4 days before and ending 4 days after the day in question. The time spent in an interruption will be defined as the proportion of days when the running average was less than or equal to 10%.[48]

ix. Engagement in care

1. Health care utilization: We collected data on urgent care visits and adherence to regular clinic visits using both self-report and abstraction of data from medical records comparing their scheduled visit date with their actual visit date. This method has used to assess clinic attendance in the literature.[49,50]
2. Competing demands: Questions were modified from Gelberg and Anderson's Behavioral Model for Vulnerable Populations [51,52] to assess how often lack of food interferes with ability to procure drugs or visit the clinic.

c. Primary Aim 3 Outcomes:

i. <u>Costs per person</u>: Including recruitment, training, support, and loan administration and monitoring (pumps and other commodities will be purchased by participants, thus not a direct program cost). Loans and their repayment will be tracked. We will measure costs using program expenditure records, explained as necessary by the program manager, and focused "time and motion" studies to allocate staff time across tasks within and outside the intervention. Costs will be classified by program activity and by standard resource

categories (e.g., personnel, supplies, services). Costs will also be classified as experienced by the program (e.g., recruitment and training) and by partners (e.g., microfinance agency) that operate without program subsidy. Donated and subsidized resources will be appraised at market value. Because program implementation is standard across sites and centrally managed, cost data collection will be efficient.

ii. Net costs: Program costs adjusted for added or averted health care costs. We will base changes in short-term health care costs on household surveys (household expenditures for health care for illness episode and hospitalizations). Longer-term health care costs will be projected using clinical simulation modeling, based on observed changes in health status (e.g. HIV morbidity), combined with estimates from the trial and published studies of the costs of managing these conditions. Projections of HIV health care costs are imprecise but essential for a full cost portrayal. Uncertainty in this measure will be explored with sensitivity analyses.

iii. Health effects: Health effects will be quantified in two ways. First, we will inventory major health-related events (deaths, detectable viral loads, hospitalizations, and opportunistic infections).

iv. Disability adjusted life years (DALYs): Second, and following best practices in CEA, we will integrate the health impact of averted adverse events using disability-adjusted life years (DALYs), including lost years of life and the collective disability effects of all the adverse events. DALY estimates will be for the short term (during the trial) and the long term (5, 10, and 20 years) using the clinical modeling.

v. Cost-effectiveness ratios: Net cost per death averted (if a significant difference is observed by study arm) and per major adverse health event averted and 2) net cost per DALY averted. Projected health and financial effects will be discounted at 3% per year. We will conduct extensive sensitivity analyses on these ratios. Importantly, if the intervention yields net savings (i.e., negative net costs) as well as health benefits, the intervention is classified as “dominant” and no CE ratio is calculated. We will conduct sensitivity analyses (one-way, two-way, scenario, and multi-way/stochastic/ Monte Carlo) to assess how results change as a function of uncertainty in input values.

28) **Analysis methods:** Analysis methods to be used and how the treatment effects will be presented:

a. *Aim 1, Hypothesis 1. The intervention will lead to improved viral load suppression (primary outcome) and other measures of morbidity (secondary outcomes) in the intervention arm compared to the control arm.* Intent-to-treat analyses will assess whether the intervention will result in improved changes in primary and secondary outcomes with mixed (i.e., fixed and random) effects, maximum likelihood models that use all of the longitudinal data and account for pair matching (if justified) and variability among clusters and individuals.[8,53] These mixed models are equivalent to repeated-measures models. Pairs, arms, visits, and the interactions between arms and visits will be the fixed effects and clusters and individuals will be the random effects. Since there are five visits, 4 degrees of freedom are used to compare each follow-up visit to the first visit. Correspondingly, four interaction terms will estimate the difference between arms in changes from visit 1 to each follow-up visit. The design included matching, so pairs should be examined in the analysis. If accounting for pairs does not explain sufficient variability to justify inclusion, which is likely because of the longitudinal data in which each individual or household

serves as their own control, pairs will be dropped from the model to preserve degrees of freedom. Since the intervention vs. control assignment was at the facility level, the analysis provides 56 (= 14 x 4) denominator degrees of freedom for t-tests of coefficients estimated in the mixed models if pairs are not accounted and 28 (= 7 x 4) denominator degrees of freedom if they are accounted. P-values will be corrected for the denominator degrees of freedom if software does not use the correct degrees of freedom. Linear contrasts among the arm x visit interaction terms will be used to estimate intervention impact for outcomes. For the primary outcome of viral load suppression, the linear contrast will be the interaction of arm with visit 1 and 5 only, thus estimating the difference between arms in the change from visit 1 to visit 5. For other primary outcomes, the linear contrast will be the interaction of arm and the trend over visits, thus estimating the difference between arms in trend (i.e., change) over visits.

b. *Aim 2, Hypothesis 2. The intervention will improve food security, increase per-capita value of food consumption, and increase household agricultural income, which in turn will contribute to improved outcomes through nutritional (improved diet quality, nutritional status), behavioral (improved ART adherence and retention in care), mental health (improved mental health/less depression) and empowerment (gender role attitudes, household decision-making) paths*[54]. We will assess direct and indirect intervention effects using structural equation modeling to examine paths from the intervention through baseline-to-endline changes in mediating outcomes to changes in primary health outcomes.[55,56] Statistical mediation will be assessed using causal inference methods of Valeri and Vanderweele,[57] which yields optimal estimates of indirect effects in the presence of non-continuous outcomes, interactions, and clustered data.
c. Any adjustment for covariates: Randomization should yield equivalence between arms on covariates, but if non-equivalence is found on baseline measures or from differential attrition, we will control for it by including the covariate in the model.
d. Methods used for assumptions to be checked for statistical methods: Distributional assumptions for outcome variables will be checked using descriptive statistics. For mediation models, the assumption of no interaction between exposure (i.e., intervention vs. control) and mediators will be checked using product terms.
e. Transformations will be used for continuous outcome variables that are skew to obtain more symmetric distributions.
f. Any planned sensitivity analyses for each outcome, where applicable: Not applicable.
g. Any planned subgroup analyses for each outcome including how subgroups are defined: *Aim 3. Identification of individuals most likely to benefit from the intervention:* We will undertake regression analyses to provide guidance on targeting of the *Shamba Maisha* intervention to subpopulations most likely to benefit. The benefits of the intervention may be realized to a greater degree by individuals with entrepreneurial ability and risk-taking preferences, compared to individuals who use loans primarily for day-to-day expenses.[58,59] To learn which participants were most likely to benefit, we will use data collected at the time of enrollment to test for heterogeneous effects of the intervention on health outcomes, using interaction terms with individual characteristics including age, gender, baseline socio-economic status (particularly household wealth), household status, and novel measures of risk preferences[60] and entrepreneurial ability.[61]

29) Missing data

The study team employed several strategies to account for and address missing data during the *Shamba Maisha* trial period. Missing data was categorized into: 1) missed individual questions, 2) missing forms, and 3) missed visits.

a. Missed individual questions: For instances when participants missed questions as part of a validated scale, for example, the Hopkins Symptoms Checklist for depression, we first assessed the amount of missingness by making a variable for the number of questions missed per participant. We checked the assumption of missing at random[62] by tabulating basic socio-demographic characteristics on questions and participants for missing values. For example, we did not impute for questions related to marriage among single or widowed participants who systematically did not answer those questions. For missing questions truly missed at random and comprising less than 25% of the total sub-scale length, however, we imputed. For questions related to alcohol consumption where non-standardized liquid portions and/or alcohol contents were reported, we used mean imputation to report drinks per day for these participants. In situations where questions that were not part of a sub-set or scale of questions were missing, these variables were left as missing. Information on percent of questions missing and imputed will be reported for each variable as appropriate in study manuscripts.

b. Missing forms: In situations where a form (i.e. part of the survey questionnaire) was missing while other data was present, the study team first examined the study register. The study register identified participants' dates for home and clinic visits. Sometimes missing data were found to be acceptable if a participant attended the home visit but did not attend the clinic visit, or vice versa. Other times, the identification of a missing form when all other forms from both home and clinic visits helped identify incorrectly entered study IDs or study visits. If after these two steps were assessed and the missing form was still unaccounted for, the study team contacted the field study manager who worked with the research assistant collecting the data to see if the data were still available on the tablet, and in such cases, resent the data to the server. Finally, if none of these steps yielded data recovery, the person-visit-form was marked as a known missing.

c. Missed visits: When a participant missed a visit, either home or clinic or both, this was noted in the study register. No data were thus imputed for this participant for that visit.

30) **Additional analyses** – None required

31) **Harms**

a. Data safety**:**

The study team took a number of proactive strategies to insure the highest levels of data safety. First, data were collected on password protected tablets. Data were collected using Open Data Kit (ODK). Further, ODK data was uploaded weekly to a secure ODK server. Data was then downloaded from the server and stored on a secure UCSF server. The data was stored in a separate folder than other study materials, and only members of the data team (Dr. Frongillo, Ms. Sheira and Mocello, and Mr. Bernard Rono, the field data manager) as well as the study director (Ms. Rachel Burger) had access to this data. Lastly, any individual who requests study data was required to sign a data agreement which included not sharing the data as well as recommendations for data safety. No identifiers have or will be shared with external investigators.

b. Details on how adverse events are coded or categorized:

Deaths were reported to KEMRI by email within 24 hours after the PIs learned of the occurrence and hard copies forwarded to SERU within three working days. Other adverse events were not reported.

Individuals were provided with information on how to contact the study staff to report adverse events associated with study participation. No adverse events were reported that were associated with study participation.

32) **Statistical software**

The following software systems will be used in the analysis of *Shamba Maisha* data: 1) SAS 9.4; 2) Stata SE version 14 [College Station, TX: StataCorp LP]; Stat Transfer 14; and likely 4) MPlus for causal mediation models since Stata can handle only one mediator at a time if causal mediation methods are needed.

33) **References**

1. van der Laan MJ, Balzer LB, Petersen ML (2012) ADAPTIVE MATCHING IN RANDOMIZED TRIALS AND OBSERVATIONAL STUDIES. J Stat Res 46: 113-156.
2. Imai K, King G, Nall C (2009) The Essential Role of Pair Matching in Cluster-Randomized Experiments, with Application to the Mexican Universal Health Insurance Evaluation. Statistical Science 24: 29-53.
3. Greevy R, Lu B, Silber JH, Rosenbaum P (2004) Optimal multivariate matching before randomization. Biostatistics 5: 263-275.
4. Lu B, Greevy R, Xu X, Beck C (2011) Optimal Nonbipartite Matching and Its Statistical Applications. Am Stat 65: 21-30.
5. Greevy RA, Jr., Grijalva CG, Roumie CL, Beck C, Hung AM, et al. (2012) Reweighted Mahalanobis distance matching for cluster-randomized trials with missing data. Pharmacoepidemiol Drug Saf 21 Suppl 2: 148-154.
6. King G, Gakidou E, Ravishankar N, Moore RT, Lakin J, et al. (2007) A "politically robust" experimental design for public policy evaluation, with application to the Mexican universal health insurance program. J Policy Anal Manage 26: 479-506.
7. Cohen CR, Steinfeld RL, Weke E, Bukusi EA, Hatcher AM, et al. (2015) Shamba Maisha: Pilot agricultural intervention for food security and HIV health outcomes in Kenya: design, methods, baseline results and process evaluation of a cluster-randomized controlled trial. Springerplus 4: 122.
8. Hayes RJ, Moulton LH (2009) Cluster Randomized Trials. Boca Raton: Chapman & Hall/CRC Press.
9. Weinberg JL, Kovarik CL (2010) The WHO clinical staging system for HIV/AIDS. AMA Journal of Ethics 12: 202-206.
10. Frongillo EA, Nanama S (2006) Development and validation of an experience-based measure of household food insecurity within and across seasons in northern Burkina Faso. J Nutr 136: 1409S-1419S.
11. Coates J, Frongillo EA, Rogers BL, Webb P, Wilde PE, et al. (2006) Commonalities in the experience of household food insecurity across cultures: what are measures missing? The Journal of nutrition 136: 1438S-1448S.
12. Miller CL, Bangsberg DR, Tuller DM, Senkungu J, Kawuma A, et al. (2010) Food insecurity and sexual risk in an HIV endemic community in Uganda. AIDS Behav.

13. Tsai A, Bangsberg D, Frongillo E, Senkungu J, Emenyonu N, et al. (2010) Gendered Effects of Food Insecurity on Depression among HIV-Infected Persons Accessing Antiretroviral Therapy (ART) in Rural Uganda. 17th Conference on Retroviruses and Opportunistic Infections. San Francisco.
14. Tsai AC, Bangsberg DR, Senkungu J, Frongillo EA, Emenyonu N, et al. Food insecurity, competing demands, and health care utilization among HIV-infected persons accessing antiretroviral therapy (ART) in rural Uganda; 2010 Feb 16-19, 2010; San Francisco, California.
15. Weiser S, Tsai A, Senkungu J, Emenyonu N, Kawuma A, et al. (2010) The impact of low sexual relationship power on viral load suppression among women receiving antiretroviral therapy in Mbarara, Uganda. 17th Conference on Retroviruses and Opportunistic Infections. San Francisco.
16. Tsai AC, Bangsberg DR, Frongillo EA, Senkungu J, Emenyonu N, et al. (2010) HIV-related stigma and food insecurity among men and women accessing ART in rural Uganda. XVIII International AIDS Conference. Vienna, Austria.
17. Weiser S, Tsai A, Senkungu J, Emenyonu N, Kawuma A, et al. (2010) Food Insecurity and Morbidity among HIV-Infected Persons Accessing Antiretroviral Therapy (ART) in Rural Uganda. XVIII International AIDS Conference. Vienna, Austria.
18. Tsai A, Gupta R, Frongillo E, Kawuma A, Senkugu J, et al. (2011) Food insecurity is associated with morbidity and patterns of healthcare utilization among HIV-infected individuals in rural Uganda. AIDS In press.
19. Hoddinott J, Yohannes Y (2002) Dietary diversity as a household food security indicator. Washington, D.C.: Food and Nutrition Technical Assistance Project, Academy for Educational Development.
20. (2015) The 2014 Kenya Demographic and Health Survey Nairobi, Kenya Kenya National Bureau of Statistics.
21. (1995) Physical Status: The Use and Interpretation of Anthropometry. Report of a WHO Expert Committee. WHO Technical Report Series 854. Geneva, Switzerland: World Health Organization.
22. Collins C, Duffield A, Myatt M (2000) Adults Assessment of Nutritional Status in Emergency-Affected Populations Geneva, Switzerland: United Nations, Administrative Committee on Coordination, Sub-committee on Nutrition. 1-28 p.
23. James WP, Ferro-Luzzi A, Waterlow JC (1988) Definition of chronic energy deficiency in adults. Report of a working party of the International Dietary Energy Consultative Group. Eur J Clin Nutr 42: 969-981.
24. Ferro-Luzzi A, Sette S, Franklin M, James WP (1992) A simplified approach of assessing adult chronic energy deficiency. Eur J Clin Nutr 46: 173-186.
25. Doocy S, Teferra S, Norell D, Burnham G (2005) Credit program outcomes: coping capacity and nutritional status in the food insecure context of Ethiopia. Soc Sci Med 60: 2371-2382.
26. Wu AW, Rubin HR, Mathews WC, Ware JE, Jr., Brysk LT, et al. (1991) A health status questionnaire using 30 items from the Medical Outcomes Study. Preliminary validation in persons with early HIV infection. Med Care 29: 786-798.
27. Chatterton ML, Scott-Lennox J, Wu AW, Scott J (1999) Quality of life and treatment satisfaction after the addition of lamivudine or lamivudine plus loviride to zidovudine-containing regimens in treatment-experienced patients with HIV infection. Pharmacoeconomics 15 Suppl 1: 67-74.
28. Mast TC, Kigozi G, Wabwire-Mangen F, Black R, Sewankambo N, et al. (2004) Measuring quality of life among HIV-infected women using a culturally adapted questionnaire in Rakai district, Uganda. AIDS Care 16: 81-94.
29. Derogatis LR, Lipman RS, Rickels K, Uhlenhuth EH, Covi L (1974) The Hopkins Symptom Checklist (HSCL). A measure of primary symptom dimensions. Mod Probl Pharmacopsychiatry 7: 79-110.
30. Bolton P, Wilk CM, Ndogoni L (2004) Assessment of depression prevalence in rural Uganda using symptom and function criteria. Soc Psychiatry Psychiatr Epidemiol 39: 442-447.
31. Bradley KA, DeBenedetti AF, Volk RJ, Williams EC, Frank D, et al. (2007) AUDIT-C as a brief screen for alcohol misuse in primary care. Alcohol Clin Exp Res 31: 1208-1217.

32. Kalichman SC, Simbayi LC, Cloete A, Mthembu PP, Mkhonta RN, et al. (2009) Measuring AIDS stigmas in people living with HIV/AIDS: the Internalized AIDS-Related Stigma Scale. AIDS Care 21: 87-93.
33. Wolfe WR, Weiser SD, Leiter K, Steward WT, Percy-de Korte F, et al. (2008) The impact of universal access to antiretroviral therapy on HIV stigma in Botswana. Am J Public Health 98: 1865-1871.
34. Weiser SD, Leiter K, Bangsberg DR, Butler LM, Percy-de Korte F, et al. (2007) Food insufficiency is associated with high-risk sexual behavior among women in Botswana and Swaziland. PLoS Med 4: 1589-1597; discussion 1598.
35. Tsai A, Bangsberg D, Kegeles S, Katz I, Muzoora C, et al. Internalized stigma, disease progression, and serostatus disclosure among people living with HIV/AIDS in rural Uganda. Annals of Behavioral Medicine: May 21, 2013 [Epub ahead of print].
36. Pronyk PM, Hargreaves JR, Kim JC, Morison LA, Phetla G, et al. (2006) Effect of a structural intervention for the prevention of intimate-partner violence and HIV in rural South Africa: a cluster randomised trial. Lancet 368: 1973-1983.
37. Kim JC, Watts CH, Hargreaves JR, Ndhlovu LX, Phetla G, et al. (2007) Understanding the impact of a microfinance-based intervention on women's empowerment and the reduction of intimate partner violence in South Africa. American journal of public health 97: 1794-1802.
38. Pulerwitz J, Gortmaker SL, DeJong W (2000) Measuring sexual relationship power in HIV/STD research. Sex Roles 42: 637-660.
39. Dunkle KL, Jewkes RK, Brown HC, Gray GE, McIntryre JA, et al. (2004) Gender-based violence, relationship power, and risk of HIV infection in women attending antenatal clinics in South Africa. Lancet 363: 1415-1421.
40. Jewkes RK, Dunkle K, Nduna M, Shai N (2010) Intimate partner violence, relationship power inequity, and incidence of HIV infection in young women in South Africa: a cohort study. Lancet 376: 41-48.
41. O'Neil JM, Denke R (2016) An empirical review of gender role conflict research: New conceptual models and research paradigms.
42. Oyugi J, Byakika-Tusiime J, Charlebois E, Kityo C, Mugerwa R, et al. (2004) Multiple validated measures of adherence indicate high levels of adherence to generic HIV antiretroviral therapy in a resource-limited setting. J Acquir Immune Defic Syndr 36: 1100-1102.
43. Giordano TP, Guzman D, Clark R, Charlebois ED, Bangsberg DR (2004) Measuring adherence to antiretroviral therapy in a diverse population using a visual analogue scale. HIV Clin Trials 5: 74-79.
44. Kelly JD, Hubenthal EA, Lurton G, Empson SF, Barrie MB, et al. (2013) Multiple self-report measures of antiretroviral adherence correlated in Sierra Leone, but did they agree? Int J STD AIDS 24: 931-937.
45. Thirumurthy H, Siripong N, Vreeman RC, Pop-Eleches C, Habyarimana JP, et al. (2012) Differences between self-reported and electronically monitored adherence among patients receiving antiretroviral therapy in a resource-limited setting. AIDS 26: 2399-2403.
46. Farley J, Hines S, Musk A, Ferrus S, Tepper V (2003) Assessment of adherence to antiviral therapy in HIV-infected children using the Medication Event Monitoring System, pharmacy refill, provider assessment, caregiver self-report, and appointment keeping. J Acquir Immune Defic Syndr 33: 211-218.
47. Lyimo RA, van den Boogaard J, Msoka E, Hospers HJ, van der Ven A, et al. (2011) Measuring adherence to antiretroviral therapy in northern Tanzania: feasibility and acceptability of the Medication Event Monitoring System. BMC Public Health 11: 92.
48. Musinguzi N, Castillo-Mancilla J, Morrow M, Byakwaga H, Mawhinney S, et al. (2019) Antiretroviral Therapy Adherence Interruptions Are Associated With Systemic Inflammation Among Ugandans Who Achieved Viral Suppression. JAIDS Journal of Acquired Immune Deficiency Syndromes 82: 386-391.

49. Evans D, Menezes C, Mahomed K, Macdonald P, Untiedt S, et al. (2013) Treatment outcomes of HIV-infected adolescents attending public-sector HIV clinics across Gauteng and Mpumalanga, South Africa. AIDS Res Hum Retroviruses 29: 892-900.
50. Hassan AS, Fielding KL, Thuo NM, Nabwera HM, Sanders EJ, et al. (2012) Early loss to follow-up of recently diagnosed HIV-infected adults from routine pre-ART care in a rural district hospital in Kenya: a cohort study. Trop Med Int Health 17: 82-93.
51. Gelberg L, Andersen RM, Leake BD (2000) The Behavioral Model for Vulnerable Populations: application to medical care use and outcomes for homeless people. Health Serv Res 34: 1273-1302.
52. Gelberg L, Gallagher TC, Andersen RM, Koegel P (1997) Competing priorities as a barrier to medical care among homeless adults in Los Angeles. Am J Public Health 87: 217-220.
53. Paul J. Gertler, Sebastian Martinez, Patrick Premand, Laura B. Rawlings, Vermeersch. CMJ (2011) Impact Evaluation in Practice. Washington DC: The International Bank for Reconstruction and Development / The World Bank.
54. Weiser SD, Young SL, Cohen CR, Kushel MB, Tsai AC, et al. (2011) Conceptual framework for understanding the bidirectional links between food insecurity and HIV/AIDS. Am J Clin Nutr 94(suppl): 1729S–1739S.
55. Bollen KA (1989) Structural Equations with Latent Variables: John Wiley & Sons, Inc.
56. Petersen ML, Sinisi SE, van der Laan MJ (2006) Estimation of direct causal effects. Epidemiology 17: 276-284.
57. Valeri L, Vanderweele TJ (2013) Mediation analysis allowing for exposure-mediator interactions and causal interpretation: theoretical assumptions and implementation with SAS and SPSS macros. Psychol Methods 18: 137-150.
58. Morduch J (1999) The microfinancne promise. Journal of Economic Literature 37: 1569 - 1614.
59. Morduch J, Karlan D (2009) "Access to finance" In Dani Rodrik and Mark Rosenzweig, eds Handbook of Development Economics. Amsterdam: Elsevier.
60. Eckel CC, Grossman PJ (2008) Forecasting risk attitudes: An experimental study using actual and forecast gamble choices. Journal of Economic Behavior & Organization 68: 1-17.
61. de Mel S, McKenzie D, Woodruff C (2008) Returns to Capital in Microenterprises: Evidence from a Field Experiment. . Quarterly Journal of Economics 123: 1329-1372.
62. Sterne JA, White IR, Carlin JB, Spratt M, Royston P, et al. (2009) Multiple imputation for missing data in epidemiological and clinical research: potential and pitfalls. Bmj 338: b2393.

Appendix:

a. **Appendix 1: Description of the intervention components**

i. *Agricultural training*: Intervention participants were also provided eight separate 3-hour training modules over the agricultural season (didactic sessions and practical demonstrations) in sustainable farming techniques, including seed selection, soil and water conservation, fertilization and crop rotation, integrated pest and disease management (IPM), pre and post-handling and marketing, and identifying improved market access for selling horticultural products. Participants were encouraged to grow locally available and environmentally sustainable crops, and to diversify crops to ensure a diverse diet and adequate markets for their produce. Participants in the intervention arm received training on the use of the MoneyMaker irrigation pump. All trainings were delivered by one of the agricultural trainers or the Study Manager, Elly Weke, who has a M.S. in Integrated Water Resources Management and a BSc in Horticulture. All trainings took place on participant's farms or a nearby location. Agricultural trainers also conducted visits to individual farms as needed to support study participants on various topics including pest management. We developed initial field-based trainings based on a needs assessment conducted prior to launching the R34 pilot, and tested and refined the training course during the pilot with support from KickStart International and a Postdoctoral Fellow from University of California Davis College of Agriculture and Environmental Sciences. Trainings were further updated based on process evaluation findings from our R34 study, and were tailored to the needs of farmers based on crop selection. Findings from the R34 also influenced our selection of the irrigation pump, the Money Maker Max Treadle pump instead of the smaller hip pump that some farmers found challenging to operate.

1. Based on our in-depth process evaluation from our pilot study, the following formal agricultural trainings were offered:
    a. Introduction to farming as a business
    b. Seed selection and vegetable nursery preparation
    c. Preparation for vegetable production
    d. Irrigation/Treadle Pump Use and Maintenance
    e. Insect Pest Identification and Management
    f. Vegetable Disease Identification and Management
    g. Developing Your Savings
    h. Farm Record Keeping

ii. *Financial training*: The intervention arm received training at baseline and at key intervals coordinated with harvesting seasons on financial management, group formation and management, record keeping, micro enterprise development, market planning and research, customer relations, preparation of a business plan, and marketing skills. Trainings were facilitated by Equity Bank in collaboration with our study team. Equity Bank developed a financial training curriculum aimed at helping farmers in developing financial skills in managing their personal money and income from their farm business. This curriculum encouraged the farmers to embrace farming as a business, use new

technologies in farming and work towards value added within the farm business. All trainings took place on participants' farms or a nearby location.

1. Two formal financial training sessions were offered:
    a. Group Dynamics
    b. Financial Management
        1. Budgeting
        2. Savings
        3. Banking services
        4. Debt Management
2. Make-up and informal sessions were also noted. This included at one site Financial Literacy, which appeared not to have been offered at any other sites.

Signature Page

Declaration

I have reviewed and agree to the Statistical Analysis Pan as presented in this document.

DocuSigned by: Sheri Weiser
E08E461595594C2...

______________________ 6/22/2020

Sheri D. Weiser, Principal Investigator Date

DocuSigned by: Craig Cohen
05249986608D499...

______________________ 6/10/2020

Craig R. Cohen, Principal Investigator Date

DocuSigned by: Elizabeth Anne Bukusi
338508516DBB492...

______________________ 6/11/2020

Elizabeth A. Bukusi, Principal Investigator Date

DocuSigned by: EDWARD FRONGILLO
46DF09E0204142D...

______________________ 6/23/2020

Edward A. Frongillo, Senior Statistician Date

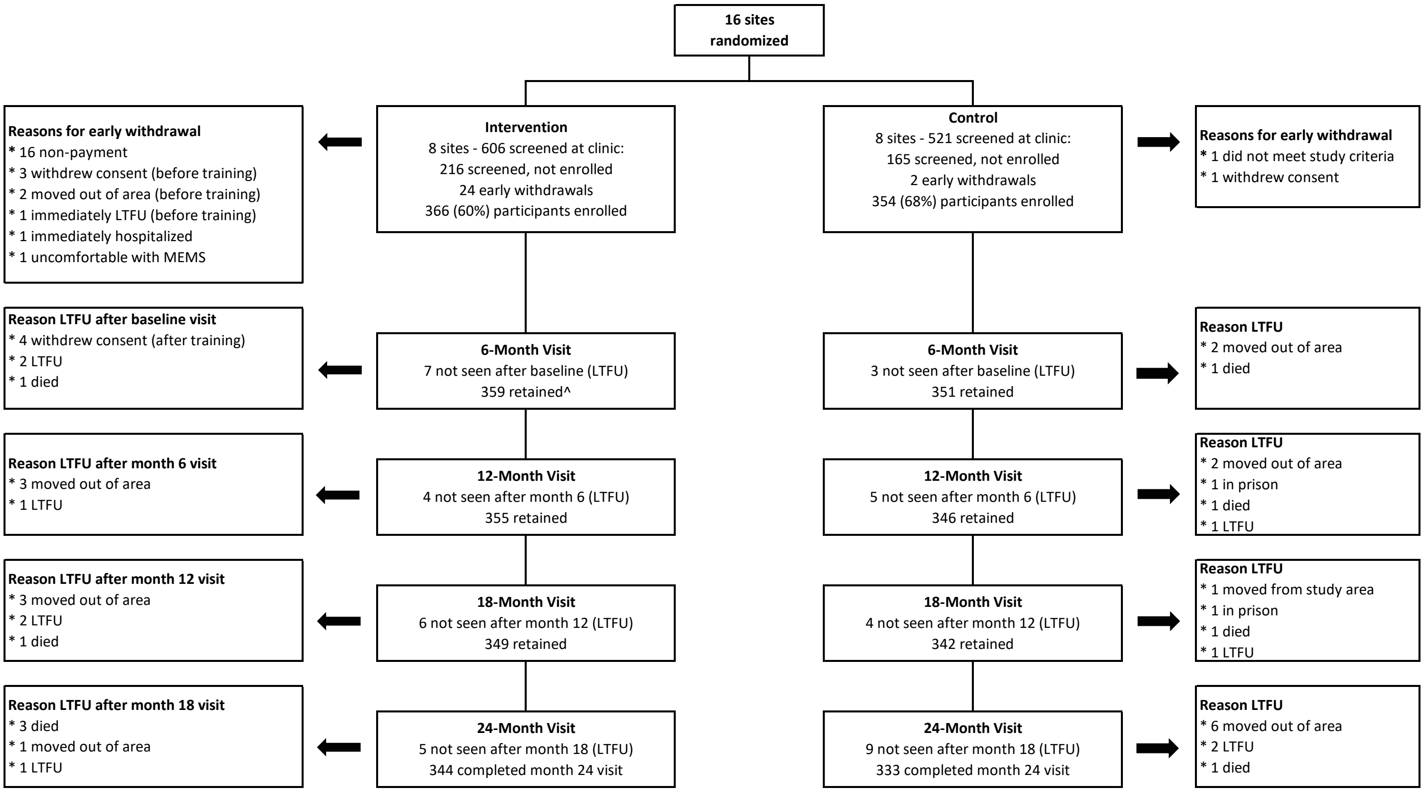

^ "Retained" means that a participant had a visit <u>on or AFTER</u> this timepoint. So, some participants retained at month 6 did not attend their month 6 visit, but did attend a later visit.
