# Supplementary material for: Effect of a Multisectoral Agricultural Intervention on HIV Health Outcomes Among Adults in Kenya: A Cluster Randomized Clinical Trial
Source: JAMA Netw Open. 2022 Dec 12;5(12):e2246158. doi: 10.1001/jamanetworkopen.2022.46158 (PMC9856331; doi:10.1001/jamanetworkopen.2022.46158)
Supplement: Supplement 2. — eMethods. Additional Data Collection and Analysis eTable 1. Viral Load Readiness Assessment Score Among 8 Pairs of Intervention and Control Facilities eTable 2. Proportion of Participants With an HIV RNA Viral Load <200 Copies/mL at Baseline and Using Dolutegravir-Based Antiretroviral Therapy at the 24-Month Follow-up Visit Among the 8 Pairs of Intervention and Control Facilities eTable 3. Main Outcomes Among Male Participants eTable 4. Main Outcomes Among Female Participants [file jamanetwopen-e2246158-s002.pdf]

Supplementary Online Content

Cohen CR, Weke E, Frongillo EA, et al. Effect of a multisectoral agricultural intervention on HIV health outcomes among adults in Kenya: a cluster randomized clinical trial. *JAMA Netw Open*. 2022;5(12):e2246158. doi:10.1001/jamanetworkopen.2022.46158

eMethods. Additional Data Collection and Analysis
eTable 1. Viral Load Readiness Assessment Score Among 8 Pairs of Intervention and Control Facilities
eTable 2. Proportion of Participants With an HIV RNA Viral Load <200 Copies/mL at Baseline and Using Dolutegravir-Based Antiretroviral Therapy at the 24-Month Follow-up Visit Among the 8 Pairs of Intervention and Control Facilities
eTable 3. Main Outcomes Among Male Participants
eTable 4. Main Outcomes Among Female Participants

This supplementary material has been provided by the authors to give readers additional information about their work.

© 2022 Cohen CR et al. *JAMA Network Open*.

eMethods. Additional Data Collection and Analysis

Method Viral Load Scale-Up Clinical Facility Readiness Tool
We conducted a Viral Load (VL) Scale-Up Clinical Facility Readiness Assessment developed by ICAP at Colombia University at the 16 health facilities participating in the *Shamba Maisha* study. For the assessment, we focused on clinical care-related questions relevant to interpretation and HIV management of adults. This assessment scored the facility based on its readiness to provide routine viral load monitoring for patients on ART, including assessing clinical systems in place for implementation of routine VL testing and interpretation. The assessment included 36 questions in the following categories: 1) Type of Testing, Testing Algorithms, and Staff Responsibilities 2) Pre-testing, 3) Post-Testing, 4) Enhanced Adherence Testing, and 5) Managing Virologic Failure. Facilities were scored Yes (1), Partial (0.5), or No (0), where applicable. For example, for the following question: Are patients receiving their results in a specified time period? If yes, score based on average period of time: Within 2 weeks (score=yes), 2-6 weeks (score=partial), >6 weeks (score=no).

Agricultural Training Details
Topics covered included sustainable farming techniques including use of regenerative agriculture to replete the soil of essential depleted nutrients, use and repair of the pump (replacement parts are available at most local agrovet shops and no tools are required to install them), seed selection, plant spacing, soil and water conservation, integrated pest and disease management, pre- & post-harvest handling and marketing, record keeping, savings, investments, and group dynamics (overview of training curriculum: https://shambamaisha.ucsf.edu/events/agriculture-training). Importantly, participants were trained in sustainable agriculture techniques, helping to improve soil fertility and productivity while reducing reliance on inorganic fertilizers and pesticides. Individuals in the control arm were offered a similar intervention at the conclusion of their participation in the trial.

Enrollment of Participants: enrichment of non-suppressed participants
Due to the high proportion of viral suppression among participants in the first four facilities, for the remaining study sites we preferentially attempted to recruit 30% of participants per community who were at higher risk for viral non-suppression. We did this by reviewing medical records to identify individuals who in the past 12 months had either 1) a detectable viral load, 2) ART interruption or fair-to-poor ART adherence, or 3) at least one missed visit by more than three days.

Sample Size Calculation
Data from the *Shamba Maisha* pilot study were used to estimate the sample size for the key outcomes: changes from month 0 to month 24 in viral load suppression (primary outcome variable), and in food insecurity score (key mediating variable). We assumed that the standard deviation (SD) values in this study would be similar to those in the pilot study because the populations had similar geographic and demographic characteristics.[24] To be conservative, we assumed a coefficient of variation due to clustering of 0.150, ignoring the matched pairs. [29] Retention in the pilot study was 98%. To be conservative, ≥90% retention was assumed for this study. Assuming, as in our pilot study, that 0.150 of the sample in the control arm became suppressed from baseline to month 24, there would be 80% power to detect differences such that the proportion becoming suppressed from baseline to month 24 in the intervention arm would be ≥0.288. With regards to two secondary outcomes, the within-arm SD for changes in food insecurity score and in CD4 cell count were estimated from the pilot study as 2.95 and 208.9 cells/mm^3, respectively. The sample size provided 80% power to detect a difference of 1.2 for food insecurity (HFIAS score) and ≥57 cells/mm^3 for CD4 count. No interim analyses or stopping guidelines related to sample size were necessary for the trial.

Randomization and Site Selection
Based on the pilot study results, we chose minimum requirements for health facility inclusion in the study as: Ministry of Health facility that served a minimum of 350 patients on ART; proximity to one or more permanent water source (e.g., river, lake, stream, shallow aquifers that can be reached with hand-dug wells); arable land; proximity to markets; and farming as a key economic activity in the community (i.e., ≥50% of the population involved in agriculture as the primary means of income, which applied to all of the Ministry of Health facilities in

© 2022 Cohen CR et al. *JAMA Network Open*.

the Nyanza Region). These criteria led to the identification of 36 eligible health facilities, of which we selected 16 facilities comprised of eight well-matched pairs based on facility type (i.e., subcounty hospital, health center, or dispensary), geography defined by sub-county, soil type, primary source of water for irrigation (i.e., lake, river/stream, shallow wells), and access to markets. The health facilities that were matched on the above characteristics were randomized within pairs to the intervention or control arms using random numbers computer-generated by the study biostatistician.

Results
The viral load scale-up clinical facility readiness tool was used to calculate a score (maximum 36 points) for each intervention and control facility. For intervention facilities, the score ranged from 26.5 to 32.5 (73.6% to 90.3%) and from 27.0 to 34.0 (75.0% to 94.4%) for control facilities, with no apparent difference between the eight pairs of intervention and control facilities. Dolutegravir roll-out in Kenya commenced in early 2018. The first use in a study participant was reported at the 12-month visit. By the endline visit at month 24, dolutegravir-containing ART was reported by 3% to 51% of participants across the eight intervention facilities and 3% to 49% of participants across the eight control facilities.

© 2022 Cohen CR et al. *JAMA Network Open*.

eTable 1. Viral Load Readiness Assessment Score Among 8 Pairs of Intervention and Control Facilities

No.	Facility	(I)ntervention (C)ontrol Site	Type of Testing, Testing Algorithms, & Staff Responsibilities	Pre-Test	Post-Test	Enhanced Adherence Counseling	Managing Virologic Failure	Total Score
	No. of questions		10	6	12	3	5	36
1	Lumumba	I	100%	75%	83%	100%	100%	90.3%
2	KCH	C	100%	100%	83%	100%	100%	94.4%
3	Pandi	I	100%	67%	63%	100%	100%	81.9%
4	Railways	C	90%	67%	63%	67%	100%	76.4%
5	Honga Ogosa	C	90%	50%	88%	100%	90%	83.3%
6	Nyangande	I	100%	83%	75%	100%	90%	87.5%
7	Sindo	I	80%	50%	71%	100%	100%	76.4%
8	Kitare	C	90%	75%	83%	50%	100%	83.3%
9	Minyenya	I	90%	58%	54%	100%	100%	75.0%
10	Ngodhe	C	100%	33%	67%	100%	100%	77.8%
11	Oyani	C	90%	42%	63%	100%	100%	75.0%
12	Nyamasare	I	90%	50%	54%	100%	100%	73.6%
13	Sori Lakeside	I	100%	83%	71%	100%	100%	87.5%
14	Muhuru Bay	C	100%	83%	63%	100%	100%	84.7%
15	Osingo	C	100%	50%	63%	100%	100%	79.2%
16	Suna Ragana	I	75%	75%	58%	100%	100%	75.0%

© 2022 Cohen CR et al. *JAMA Network Open*.

eTable 2. Proportion of Participants With an HIV RNA Viral Load <200 Copies/mL at Baseline and Using Dolutegravir-Based Antiretroviral Therapy at the 24-Month Follow-up Visit Among the 8 Pairs of Intervention and Control Facilities

No.	Facility	Intervention vs. Control	% Participants with a HIV RNA Viral Load <200 copies/mL at baseline	% of Participants on a Dolutegravir-based Regimen at endline
1	Lumumba	Intervention	89.2	2.9
2	KCH	Control	100.0	13.6
3	Pandi	Intervention	83.4	6.4
4	Railways	Control	97.7	2.5
5	Nyangande	Intervention	89.5	7.1
6	Honga Ogosa	Control	82.0	25.6
7	Sindo	Intervention	82.0	23.9
8	Kitare	Control	65.1	48.8
9	Minyenya	Intervention	91.4	47.8
10	Ngodhe	Control	89.5	33.3
11	Nyamasare	Intervention	70.4	37.5
12	Oyani	Control	95.8	31.7
13	Sori Lakeside	Intervention	81.8	51.2
14	Muhuru Bay	Control	86.7	47.7
15	Suna Ragana	Intervention	80.0	44.0
16	Osingo	Control	88.2	42.9

© 2022 Cohen CR et al. *JAMA Network Open*.

eTable 3. Main Outcomes Among Male Participants

	**Control** (n = 160)		**Intervention** (n = 164)		**Trend per 24 months**		**Difference in trend between arms**	**95% CI**	**p-value**
	**Visit 1**	**Visit 5**	**Visit 1**	**Visit 5**	**Control**	**Intervention**			
	N (%) or Median (IQR)		N (%) or Median (IQR)						
**HIV Outcomes**									
% Virally suppressed (≤200 copies/mL)	140 (87.5)	141 (94.6)	142 (86.6)	145 (96.7)	1.01	2.00	0.99*	(-0.22, 2.20)	0.11
CD4 (≤500 cells)	87 (54.4)	75 (50.3)	78 (47.6)	69 (46.0)	-0.34	-0.05	0.39	(-0.49, 1.27)	0.38
% Hospitalized in the past 6 months	12 (7.5)	4 (2.7)	8 (4.9)	5 (3.3)	-0.91	-0.58	0.32	(-1.28, 1.93)	0.69
% with AIDS-defining condition	8 (5.0)	1 (0.7)	11 (6.7)	2 (1.3)	-2.24	-1.67	0.57	(-1.92, 3.06)	0.65
**Food Insecurity**									
Food insecurity score (0-27, higher score = more insecure)	19.5 (17.0, 23.0)	15.0 (14.0, 19.0)	22.0 (19.0, 25.0)	14.0 (11.0, 18.0)	-3.99	-7.75	-3.76	(-4.71, -2.81)	< 0.001
Food insecurity (categorical)									
Food secure	0 (0.0)	10 (6.8)	0 (0.0)	26 (17.5)					
Mildly food insecure	2 (1.2)	8 (5.4)	0 (0.0)	31 (20.8)					
Moderately food insecure	43 (26.9)	98 (66.7)	34 (20.7)	45 (30.2)					
Severely food insecure	115 (71.9)	31 (21.1)	130 (79.3)	47 (31.5)					
**Nutrition**									
Body Mass Index (kg/m2, continuous)	21.1 (19.3, 22.9)	21.4 (19.5, 22.8)	20.7 (19.4, 22.3)	21.1 (19.7, 22.9)	0.35	0.44	0.09	(-0.13, 0.31)	0.44
**Behavioral Pathway**									
% Missed scheduled HIV visit in the past 6 months	52 (32.5)	7 (4.7)	58 (35.6)	23 (15.3)	-2.32	-1.31	1.01	(0.05, 2.02)	0.04
ART Adherence (continuous, self-report)	100 (98.3, 100)	100 (100, 100)	100 (98.3, 100)	100 (100, 100)	1.17	1.14	-0.03	(-1.35, 1.29)	0.97
ART Adherence, self-report									
95-100%	149 (94.9)	144 (97.3)	155 (95.1)	146 (98.0)					
75-94%	7 (4.5)	4 (2.7)	7 (4.3)	3 (2.0)					
<75%	1 (0.6)	0 (0.0)	1 (0.6)	0 (0.0)					
**Mental Health**									
Mental health score of MOS HIV	67.3 (53.0, 75.5)	82.8 (76.3, 85.9)	62.8 (49.1, 72.7)	80.5 (64.8, 86.5)	14.78	14.88	0.10	(-3.10, 3.29)	0.95
Physical health score of MOS HIV	87.1 (83.4, 89.3)	86.4 (85.5, 87.7)	85.4 (77.7, 88.6)	86.2 (85.2, 87.3)	-1.67	3.03	4.71	(1.72, 7.70)	0.002

© 2022 Cohen CR et al. *JAMA Network Open*.

Depression score (continuous)	1.3 (1.1, 1.7)	1.1 (1.0, 1.2)	1.5 (1.2, 1.9)	1.0 (1.0, 1.2)	-0.32	-0.42	-0.11	(-0.30, 0.08)	0.28
% with Probable depression	35 (21.9)	11 (7.4)	61 (37.4)	7 (4.7)	-2.19	-3.24	-1.05	(-2.20, 0.09)	0.07
Social support score	17.0 (14.0, 20.0)	16.0 (13.0, 20.0)	16.0 (14.0, 20.0)	12.0 (11.0, 15.0)	-0.59	-4.38	-3.80	(-4.77, -2.82)	<0.001
**Empowerment**									
Self-Confidence	5.0 (4.0, 5.0)	4.0 (4.0, 5.0)	5.0 (4.0, 6.0)	4.0 (4.0, 5.0)	-0.16	-0.79	-0.63	(-0.95, -0.32)	<0.001
* Difference-in-differences estimates for detectable viral load calculated between visit 1 and visit 5 (~24 months)									

© 2022 Cohen CR et al. *JAMA Network Open*.

eTable 4. Main Outcomes Among Female Participants

	**Control** (n = 194)		**Intervention** (n = 202)		**Trend per 24 months**		**Difference in trend between arms**	**95% CI**	**p-value**
	**Visit 1**	**Visit 5**	**Visit 1**	**Visit 5**	**Control**	**Intervention**			
	N (%) or Median (IQR)		N (%) or Median (IQR)						
**HIV Outcomes**									
% Virally suppressed (≤200 copies/mL)	151 (78.2)	173 (94.0)	172 (85.2)	182 (93.8)	1.96	1.61	-0.35*	(-1.41, 0.71)	0.52
CD4 (≤500 cells)	65 (33.5)	54 (29.4)	65 (32.2)	59 (30.4)	-0.41	-0.12	0.29	(-0.52, 1.11)	0.48
% Hospitalized in the past 6 months	8 (4.2)	14 (7.6)	26 (12.9)	16 (8.3)	0.52	-0.35	-0.87	(-1.98, 0.24)	0.12
% with AIDS-defining condition	9 (4.7)	1 (0.5)	7 (3.5)	2 (1.0)	-2.14	-0.98	1.17	(-1.08, 3.41)	0.31
**Food Insecurity**									
Food insecurity score (0-27, higher=more insecure)	21.0 (18.0, 24.0)	16.0 (14.0, 19.0)	22.0 (20.0, 25.0)	14.0 (11.0, 17.0)	-4.93	-8.29	-3.36	(-4.18, -2.53)	< 0.001
Food insecurity (categorical)									
Food secure	0 (0.0)	7 (3.8)	0 (0.0)	28 (14.5)					
Mildly food insecure	0 (0.0)	18 (9.8)	1 (0.5)	41 (21.2)					
Moderately food insecure	34 (17.5)	117 (63.6)	38 (18.8)	61 (31.6)					
Severely food insecure	160 (82.5)	42 (22.8)	163 (80.7)	63 (32.6)					
**Nutrition**									
Body Mass Index (kg/m2, continuous)	21.8 (20.0, 24.8)	22.4 (20.3, 26.2)	22.5 (20.6, 25.5)	22.9 (20.9, 26.1)	0.74	0.29	-0.45	(-0.71, -0.19)	0.001
**Behavioral Pathway**									
% Missed ≥ one scheduled HIV visit in the past 6 months	55 (28.5)	13 (7.1)	83 (41.1)	37 (19.3)	-1.83	-1.06	0.78	(-0.04, 1.60)	0.06
ART Adherence (continuous, self-report)	100 (98.3, 100)	100 (100, 100)	100 (98.3, 100)	100 (100, 100)	0.002	0.21	0.21	(-0.88, 1.29)	0.71
ART Adherence, self-report									
95-100%	182 (94.8)	178 (97.3)	189 (95.0)	185 (97.9)					
75-94%	10 (5.2)	4 (2.2)	10 (5.0)	3 (1.6)					
<75%	0 (0.0)	1 (0.5)	0 (0.0)	1 (0.5)					
**Mental Health**									
Mental health score of MOS HIV	62.3 (48.7, 74.0)	77.7 (68.5, 83.1)	55.1 (43.8, 67.3)	71.4 (57.0, 81.7)	12.48	13.33	0.85	(-2.25, 3.95)	0.59
Physical health score of MOS HIV	83.6 (66.9, 88.2)	86.0 (76.6, 87.3)	82.9 (71.1, 87.2)	85.5 (77.2, 87.4)	3.40	2.18	-1.27	(-4.84, 2.30)	0.49
Depression score (continuous)	1.5 (1.2, 1.9)	1.2 (1.1, 1.5)	1.8 (1.4, 2.1)	1.2 (1.1, 1.4)	-0.22	-0.47	-0.25	(-0.47, -0.04)	0.02

© 2022 Cohen CR et al. *JAMA Network Open*.

% with Probable depression	71 (36.6)	30 (16.3)	108 (53.5)	29 (15.1)	-1.86	-2.65	-0.79	(-1.55, -0.03)	0.04
Social support score	18.0 (15.0, 22.0)	18.0 (13.5, 22.0)	18.0 (14.0, 21.0)	13.5 (11.0, 17.0)	-0.44	-3.94	-3.50	(-4.44, -2.56)	<0.001
**Empowerment**									
Self-Confidence	6.0 (4.0, 7.0)	5.0 (4.0, 7.0)	6.0 (5.0, 7.0)	4.0 (4.0, 6.0)	-0.55	-0.71	-0.16	(-0.46, 0.13)	0.28
* Difference-in-differences estimates for detectable viral load calculated between visit 1 and visit 5 (~24 months)									

© 2022 Cohen CR et al. *JAMA Network Open*.
