# Supplementary material for: Effect of a Multisectoral Agricultural Intervention on HIV Health Outcomes Among Adults in Kenya: A Cluster Randomized Clinical Trial
Source: JAMA Netw Open. 2022 Dec 12;5(12):e2246158. doi: 10.1001/jamanetworkopen.2022.46158 (PMC9856331; doi:10.1001/jamanetworkopen.2022.46158)
Supplement: Supplement 3. — Data Sharing Statement [file jamanetwopen-e2246158-s003.pdf]

Data Sharing Statement

Cohen. Effect of a Multisectoral Agricultural Intervention on HIV Health Outcomes Among Adults in Kenya. *JAMA Netw Open*. Published December 12, 2022.
doi:10.1001/jamanetworkopen.2022.46158

Data
Data available: Yes
Data types: Deidentified participant data, Data dictionary
How to access data: Requests to: craig.cohen@ucsf.edu
When available: With publication

Supporting Documents
Document types: None

Additional Information
Who can access the data: researchers whose proposed use of data has been approved by IRB
Types of analyses: Any purpose
Mechanisms of data availability: Signed data access agreement
